# Altered dynamic functional network connectivity patterns in Alzheimer’s disease: insights into neural dysfunction

**DOI:** 10.3389/fnagi.2025.1617191

**Published:** 2025-07-23

**Authors:** Xuerui Pang, Yi Ji, Chenyang Hu, Yulong Dai, Panpan Hu, Xingqi Wu, Kai Wang

**Affiliations:** ^1^Department of Neurology, The First Affiliated Hospital of Anhui Medical University, Hefei, China; ^2^Anhui Province Key Laboratory of Cognition and Neuropsychiatric Disorders, Hefei, China; ^3^Collaborative Innovation Center of Neuropsychiatric Disorders and Mental Health, Hefei, China; ^4^Institute of Artificial Intelligence, Hefei Comprehensive National Science Center, Hefei, China; ^5^School of Mental Health and Psychological Sciences, Anhui Medical University, Hefei, China

**Keywords:** Alzheimer’s disease, dynamic functional network connectivity, functional magnetic resonance imaging, independent component analysis, network

## Abstract

**Background:**

Dynamic functional network connectivity (dFNC) assesses temporal fluctuations in functional connectivity (FC) during magnetic resonance imaging (MRI), capturing transient changes in neural activity. Investigating dFNC may provide valuable insights into the complex clinical manifestations of Alzheimer’s disease (AD). However, research on dynamic FC alterations in AD remain limited. This study aimed to comprehensively characterize dFNC patterns in patients with AD.

**Methods:**

A total of 100 patients diagnosed with AD and 69 with healthy controls (HC) were included. Resting-state functional magnetic resonance imaging (rs-fMRI) data were analyzed using a sliding-window approach and k-means clustering based on independent component analysis to examine dFNC alterations. Correlation analyses were conducted to assess associations between dFNC variations and clinical scores in individuals with AD. Additionally, an exploratory multivariate pattern analysis was performed to classify AD across different dFNC states.

**Results:**

Four recurrent connectivity states were identified. In state III, patients with AD exhibited a significantly longer mean dwell time and a higher fractional time compared to the HC group, whereas the opposite trend was observed in state IV. In state III, both fractional time and mean dwell time were negatively correlated with cognitive scores. Significant differences in FC strength were observed between states II and III. The highest classification accuracy for distinguishing AD was achieved in state II, which was characterized by intra- and inter-network dysfunction across multiple functional networks.

**Conclusion:**

Distinct alterations in dFNC were identified, with significant associations observed between connectivity patterns and clinical symptoms. These findings provide new insights into the pathophysiology of AD.

## 1 Introduction

Alzheimer’s disease (AD) is a progressive neurodegenerative disorder predominantly affecting individuals over the age of 65. Globally, an estimated 44 million individuals have been diagnosed with AD, including 15.07 million individuals over the age of 60 in China (Ren et al., 2022). The condition imposes a substantial medical and economic burden on society. AD is characterized by progressive memory decline, cognitive impairment, and psychiatric symptoms; however, its underlying pathophysiological mechanisms remain incompletely understood. Proposed mechanisms include the accumulation of extracellular β-amyloid deposition, abnormal intracellular aggregation of hyperphosphorylated tau proteins, and neuroinflammation mediated by microglial activation (Terry, 1994; Serrano-Pozo et al., 2011; Leng and Edison, 2021; Xu et al., 2024).

Current gold-standard diagnostic approaches, such as cerebrospinal fluid analysis and positron emission tomography, are invasive and may limit patient adherence. Additionally, neuropsychiatric scale assessments add complexity and may prolong the diagnostic process. There is an urgent need for non-invasive and highly sensitive techniques to facilitate early detection, with the potential to reduce hospitalization and mortality.

Recent advancements in magnetic resonance imaging (MRI) technology have significantly improved the study of resting-state functional MRI (rs-fMRI) in AD. Findings from these studies indicate that disruptions in functional connectivity (FC) within specific brain regions and large-scale networks may contribute to the clinical manifestations of AD. Frequently observed disruptions include intra-network FC impairments within the default mode network (DMN) and inter-network FC changes involving the cognitive executive network (CEN), salience network, and attention network (Palmqvist et al., 2017; Soman et al., 2020; Brier et al., 2012; Schultz et al., 2017). Dysfunction in high-level network hubs, often referred to as “rich hubs,” may be associated with the complex cognitive and psychiatric symptoms of AD, potentially reflecting underlying neuropathological mechanisms.

Alterations in FC provide a more comprehensive understanding of brain dysfunction in AD and may serve as sensitive imaging biomarkers for early detection. While static functional network connectivity (FNC) analysis offers valuable insights into disease characteristics, dynamic functional network connectivity (dFNC) provides a more refined approach by considering the brain as a dynamic system. dFNC analysis captures time-varying data in the FC matrix during scanning, facilitating the identification of transient connectivity patterns.

Independent component analysis, a data-driven method, decomposes rs-fMRI data into functionally distinct regions, allowing for whole-brain analysis without reliance on predefined regions of interest, which may obscure or misrepresent distinct patterns (Hyvärinen and Oja, 2000). This approach has been applied to various neurological disorders, including depression (Wu et al., 2024; Yao et al., 2019), schizophrenia (Damaraju et al., 2014), Parkinson’s disease (Kim et al., 2017; Fiorenzato et al., 2019), and cerebral small-vessel disease (Chen et al., 2024), where abnormalities in the temporal properties of dynamic FC have been reported. However, research on FC dynamics in AD remain limited (Jones et al., 2012; Fu et al., 2019; Schumacher et al., 2019). Jones et al. (2012) identified changes in the non-stationary modular organization of brain networks in AD, while Fu et al. (2019) and Schumacher et al. (2019) compared FC dynamics in AD with those observed in other forms of dementia. However, these studies were constrained by small sample sizes and lacked a comprehensive characterization of dFNC properties.

This study examined dFNC alterations in individuals with AD using independent component analysis (ICA) and k-means clustering. The relationship between these alterations and cognitive as well as psychiatric symptoms were examined. Additionally, support vector machine (SVM) classification was applied to evaluate between-group differences in FC matrices across different states, providing further insights into the imaging characteristics associated with AD.

## 2 Materials and methods

### 2.1 Participants

Individuals with AD were recruited from the Neurology Outpatient Clinic at the First Affiliated Hospital of Anhui Medical University between March 2017 and July 2024. Age-, sex-, and education-matched healthy controls (HC) were selected from the local community. The diagnosis of AD was established based on the criteria set by the Neurological and Communicative Disorders and Stroke and the AD and Related Disorders Association (NINCDS-ADRDA) for probable AD (McKhann et al., 2011). Disease severity was evaluated using the Clinical Dementia Rating, with scores ranging from 0.5 to 2.

To be included in the HC group, participants were required to have no cognitive impairment or functional limitations in daily living, as indicated by a Mini-Mental State Examination (MMSE) score of ≥ 27. The exclusion criteria encompassed the following: (1) hearing impairment or uncorrected visual deficits, (2) a history of other dementia subtypes, psychiatric disorders, stroke, or significant cranial injuries, (3) a history of substance abuse, and (4) contraindications to MRI.

### 2.2 Neuropsychological and neuropsychiatric assessment

A standardized neuropsychological assessment was administered to each participant by two qualified psychologists. Cognitive functions were evaluated across five domains: (1) General cognitive performance was assessed using the MMSE and the Montreal Cognitive Assessment; (2) Episodic memory was measured using the Chinese version of the Auditory Verbal Learning Test (CAVLT), including CAVLT-immediate (CAVLT-I), CAVLT-delayed (CAVLT-D), and CAVLT-recognition (CAVLT-R); (3) Language function was evaluated with the Verbal Fluency Test (VFT); (4) Attention performance was assessed using the Digital Span Test; and (5) Visuospatial ability was examined with the Clock Drawing Test. Neuropsychiatric symptoms were assessed using the Neuropsychiatric Inventory-12 (NPI-12).

### 2.3 MRI acquisition

MRI was conducted using a 3.0T GE scanner (GE Healthcare, Buckinghamshire, United Kingdom). Functional and structural images were acquired from all participants (see Supplementary material).

### 2.4 Data preprocessing

Rs-fMRI data preprocessing was conducted using the MRI preprocessing module within the Graph Theoretical Network Analysis toolbox (version 2.0)^1^ in MATLAB (The MathWorks, Inc., Natick, MA, United States) (Wang et al., 2015). The first 10 volumes were discarded to ensure signal equilibrium. Slice acquisition followed an alternating sequence in the positive direction, beginning with odd-numbered slices. To correct for head motion, images were realigned to the mean volume.

Sequential preprocessing steps included head motion correction, nuisance signal regression (removing white matter and cerebrospinal fluid signals, as well as Friston’s 24 head motion parameters), spatial normalization to the standard Montreal Neurological Institute echo-planar imaging template, and resampling to a voxel size of 3 × 3 × 3 mm across 207 time points. A 6 mm full-width at half-maximum Gaussian kernel was applied for spatial smoothing.

Realignment parameters identified five participants with a head displacement exceeding 3.0 mm or angular rotation exceeding 3.0°, leading to their exclusion from the study. Independent two-sample *t*-tests indicated no significant difference in mean framewise displacement (Jenkinson) between the AD and HC groups (AD group: 0.066 ± 0.032; HC group: 0.063 ± 0.025; p = 0.456).

### 2.5 Group ICA

Following preprocessing, spatial group ICA was conducted to extract functional brain networks using the Infomax algorithm within the GIFT 4.0 software package^2^. Principal component analysis was applied to each participant’s data for dimensionality reduction, yielding 120 components. Data from all participants were then combined and further reduced using expectation maximization, resulting in 100 independent components (ICs). The Infomax algorithm was executed 20 times in ICASSO to enhance reliability (Bell and Sejnowski, 1995; Himberg et al., 2004). The time series and spatial distribution of ICs for each participant were obtained using the group ICA inverse reconstruction algorithm (Calhoun et al., 2001).

Physiological noise, motion artifacts, and imaging irregularities were excluded through template recognition, visual inspection, and comparison with prior studies. The inclusion criteria for ICs were as follows: (1) peak coordinates primarily located in gray matter, (2) minimal overlap with blood vessels, white matter, ventricles, and limbic regions, (3) time series predominantly composed of low-frequency signals, and (4) a high dynamic range in the time series, defined as the difference between minimum and maximum frequencies.

Prior to computing dynamic FC, additional post-processing steps were applied (Kim et al., 2017; Allen et al., 2014). (1) Detrending was performed to eliminate data drift caused by non-linear variations during scanning, such as physiological fluctuations (e.g., heart rate and respiration); (2) despiking was conducted using AFNI’s 3dDespike algorithm to remove outliers resulting from artifacts or external interference; and (3) low-pass filtering with a fifth-order Butterworth filter was applied to remove high-frequency noise above 0.15 Hz while preserving low-frequency signal components.

### 2.6 Dynamic FC calculation

FC patterns between brain regions were analyzed using a sliding time window approach within the Dynamic Functional Connectivity toolbox in GIFT software (Preti et al., 2017). A Gaussian function (σ = 3 TRs) was used to generate the sliding window, segmenting the rs-fMRI time series into 185 rectangular windows with a step size of 1 TR and a window length of 22 TRs. Previous studies have recommended window sizes ranging from 30 to 60 s to optimize the resolution of dFNC and improve the quality of correlation matrix (Allen et al., 2014).

For each sliding window, covariance values were computed for each IC pair, resulting in a 37 × 37 pairwise covariance matrix. L1 regularization was applied to the precision matrix using the graphical least absolute shrinkage and selection operator (LASSO) framework, with 100 iterations performed to enhance sparsity (Friedman et al., 2008; Smith et al., 2011). To stabilize variance before further analysis, all FC matrices were converted to z-scores using Fisher’s Z-transformation.

### 2.7 Dynamic FC state analysis

Clustering analysis was performed on the FC matrices derived from the time windows of all participants. The k-means clustering algorithm was repeated 100 times to minimize the influence of random initial cluster selections and enhance result stability. Similarities between functional connection matrices were measured using the Euclidean distance metric, and the optimal number of clusters was determined based on the elbow criterion (see Supplementary Figure S8; Damaraju et al., 2014).

The temporal properties of the four dFNC states were quantified using three metrics within the state transition vector: (1) Mean dwell time, representing the number of consecutive windows spent in a specific state; (2) Fractional windows, indicating the proportion of time spent in each state; and (3) Transition count, reflecting the frequency and reliability of state transitions. Validation analyses were conducted using window lengths of 15, 20, 25, and 30 TRs (see Supplementary Tables S2, S3 and Supplementary Figures S9–S12).

Comparisons of FC strength between the AD and HC groups were performed across all identified states. Two-sample independent *t*-tests were used to assess differences in FC strength at 666 regional pairings, with false discovery rate (FDR) correction applied (*p* < 0.05) to account for multiple comparisons.

### 2.8 Statistical methods

Clinical data analyses were performed using SPSS software (version 26.0; Chicago, IL, United States). The Kolmogorov-Smirnov test was used to assess data normality. Between-group differences in age, education, and neuropsychiatric scores were evaluated using independent two-sample *t*-tests (two-tailed), while differences in sex distribution and state proportions were analyzed using chi-square tests. Comparisons of temporal properties across states between groups were conducted using Mann-Whitney U tests.

Partial correlation analyses were used to estimate associations between clinical scores and temporal properties in individuals with AD, with FDR adjustment applied to adjust for multiple comparisons.

### 2.9 Exploratory multivariate pattern analysis

Exploratory multivariate pattern analysis, as applied in previous studies, was used to evaluate the potential of dFNC in detecting AD at the individual level (Wu et al., 2024; Liu et al., 2015; see Supplementary material).

## 3 Results

### 3.1 General and clinical data

A total of 100 individuals with AD and 69 HC were included in this study. No significant differences were observed between the groups in terms of age, sex, or education level (*p* > 0.05). Detailed demographic and clinical characteristics of both groups are presented in Table 1.

**TABLE 1:** Demographic and clinical data.

Variables	AD patients (*n* = 100)	Healthy controls (*n* = 69)	Statistics	*p*
**Demographic factors**				
Age (years)	65.37 ± 9.56	63.45 ± 7.33	*t* = 1.48	0.142
Gender (male/female)	48/52	28/41	x^2^ = 0.91	0.341
Education (years)	7.98 ± 4.78	8.42 ± 4.47	*t* = −0.60	0.550
**Psychiatric symptom**				
NPI-12	8.02 ± 11.45	1.96 ± 5.14	*t* = 4.60	0.000**
**General cognitive performance**				
MMSE	19.20 ± 4.97	28.50 ± 1.60	*t* = −14.99	0.000**
MoCA	13.32 ± 5.60	24.26 ± 3.93	*t* = −14.93	0.000**
**Memory performance**				
CAVLT-immediate	3.42 ± 2.04	8.49 ± 1.92	*t* = 16.01	0.000**
CAVLT-delay	1.33 ± 2.50	9.30 ± 2.82	*t* = 18.59	0.000**
CAVLT-recognition	10.70 ± 4.14	14.15 ± 1.06	*t* = −6.73	0.000**
**Language performance**				
VFT	9.66 ± 4.10	17.46 ± 4.03	*t* = −12.18	0.000**
**Attention performance**				
DS-forward	5.79 ± 1.41	6.86 ± 1.42	*t* = −4.83	0.000**
DS-backward	3.24 ± 1.20	4.07 ± 1.13	*t* = −4.54	0.000**
**Visual-spatial performance**				
CDT	1.73 ± 1.25	3.38 ± 1.00	*t* = −9.47	0.000**

AD, Alzheimer’s disease; NPI-12, Neuropsychiatric Inventory-12; MMSE, Mini-Mental State Examination; MoCA, Montreal Cognitive Assessment; CAVLT, Chinese version of the Auditory Verbal Learning Test; VFT, Verbal Fluency Test; DS, Digit Span Test; CDT, Clock Drawing Test. **p < 0.01.

### 3.2 Resting state networks

**FIGURE 1:**
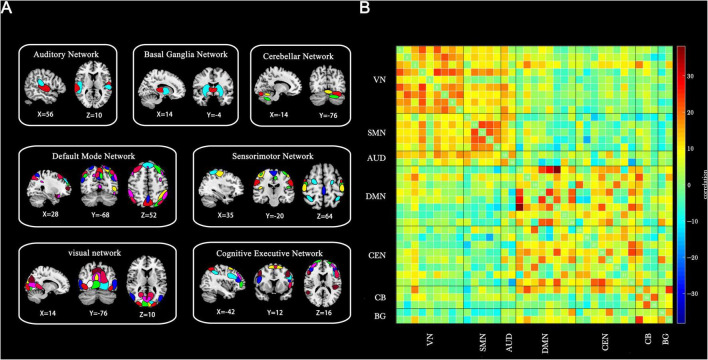
Thirty-seven ICs identified through group-independent component analysis. (A) Spatial maps of ICs categorized into seven functional brain networks based on anatomical and functional properties. (B) Group-averaged static functional connectivity between IC pairs derived from resting-state data. VN, visual network; SMN, sensorimotor network; AUD, auditory network; DMN, default mode network; CEN, cognitive executive network; CB, cerebellar network; BG, basal ganglia network.

A total of 37 ICs were selected for analysis. These components were classified into seven resting-state networks, as depicted in Figure 1 based on previous studies: the visual network (VN): ICs 7, 19, 21, 23, 36, 44, 47, 69, 81, the sensorimotor network (SMN): ICs 6, 9, 12, 17, 20, the auditory network (AUD): ICs 28, 57, the default mode network (DMN): ICs 35, 45, 50, 65, 82, 86, 93, 95, the cognitive executive network (CEN): ICs 32, 37, 46, 51, 55, 60, 76, 91, the cerebellar network (CB): ICs 8, 18, 22, and the basal ganglia network (BG): ICs 34, 59 (Preti et al., 2017). Detailed information and spatial maps of the ICs are provided in Supplementary Table S1 and Supplementary Figures S1–S7.

### 3.3 Dynamic FC state analysis

**FIGURE 2:**
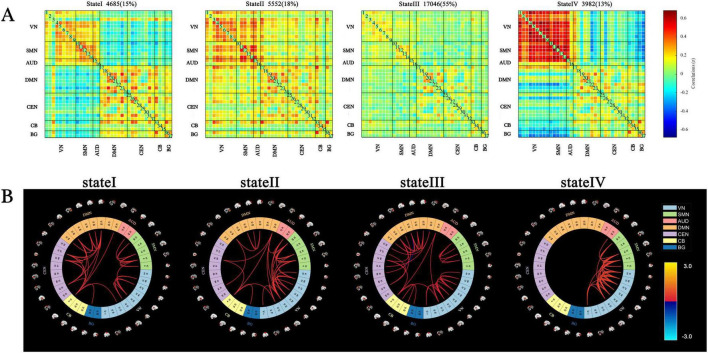
Cluster centroids and characteristics of dynamic functional network connectivity states for all participants (window length = 22 TRs). (A) Cluster centroids for each state, including the total number and percentage of occurrences (indicated in the top right of the centroid matrix). (B) Circular plots depicting the top 5% strongest connections (absolute value of the maximum correlation coefficient) in the FC matrix for each state. Each color represents one of the seven networks.

Four distinct dFNC states were identified across all participants, with the 5% strongest connections in the FC matrix for each state highlighted in Figure 2.

State I accounted for 15% of the windows and exhibited moderately positive intra-network FC within the VN, SMN, AUD, DMN, CEN, CB, and BG networks. Additionally, moderately negative inter-network FC was observed between the VN, SMN, AUD, and other networks, including the DMN, CEN, CB, and BG.

State II comprised 18% of the windows and represented a highly connected state, characterized by strong intra-network and inter-network coupling across nearly the entire brain.

State III, the most frequently occurring state at 55% of the windows, was characterized by sparse intra- and inter-network connections across most brain regions.

State IV, the least frequent state at 13% of the windows, exhibited high intra-network FC within the SMN and VN, strong inter-network FC between the SMN and VN, and notable negative correlations between the BG, VN, and SMN.

**FIGURE 3:**
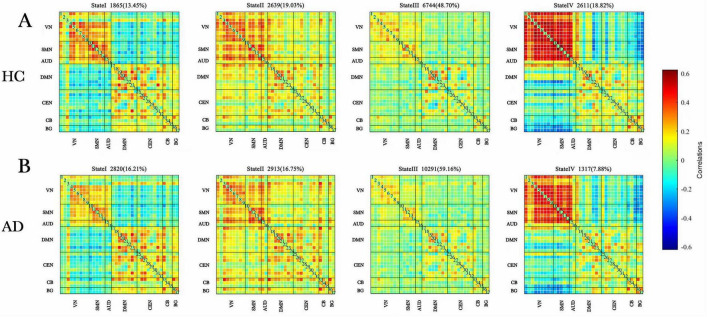
Dynamic functional connectivity patterns in the AD and HC groups. (A) Centroid matrices for the (HC) group. (B) Centroid matrices for the (AD) group. AD, Alzheimer’s disease; HC, healthy controls.

The centroid matrices for the four functional connectivity states in the AD and HC groups are presented in Figures 3A,B. Chi-square tests revealed a significant difference in the percentage of each state between the two groups (χ^2^ = 13.803, *p* = 0.003). The AD group demonstrated higher frequencies of states I and III (state I: 16.21% vs. 13.45%; state III: 59.16% vs. 48.70%), whereas states II and IV were less frequent in the AD group (state II: 16.75% vs. 19.03%; state IV: 7.88% vs. 18.82%).

**FIGURE 4:**
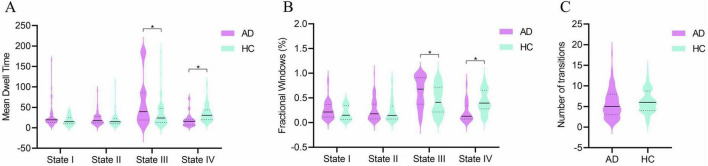
Group comparisons of temporal properties in dynamic functional connectivity states. (A) Mean dwell time in each state. (B) Fractional windows in each state. (C) Number of state transitions. Temporal properties are displayed as violin plots for the AD group (purple) and HC group (blue). Horizontal solid lines indicate the group median, while dashed lines represent the upper and lower quartiles. *p < 0.05.

Differences in the temporal properties between the two groups are depicted in Figures 4A–C. In state III, the AD group exhibited a significantly higher mean dwell time (*z* = −3.331, *p* = 0.001) and a greater proportion of fractional windows (*z* = −3.572, *p* = 0.000) compared to the HC group. Conversely, in state IV, the AD group had a significantly lower mean dwell time (*z* = −3.910, *p* = 0.000) and fewer fractional windows (*z* = −3.515, *p* = 0.000) than the HC group. No significant differences were observed between the groups in fractional windows for states I and II or in the number of state transitions (*z* = −1.626, *p* = 0.104).

### 3.4 FC strength in dynamics states

FC strength was compared between individuals with AD and HC within each state, using only the participant-specific matrix for each state, as not all participants had a corresponding window matrix for every state.

In state II, 10 stronger functional connections were observed in the AD group compared to the HC group, all of which involved inter-network connectivity, including VN-DMN, SMN-DMN, DMN-CEN, DMN-CB, CEN-CB, VN-BG, DMN-BG, and CB-BG. Additionally, one within-network connection and two between-network connections (VN, DMN-CEN, and VN-SMN) were found to be stronger in the HC group than in the AD group.

**FIGURE 5:**
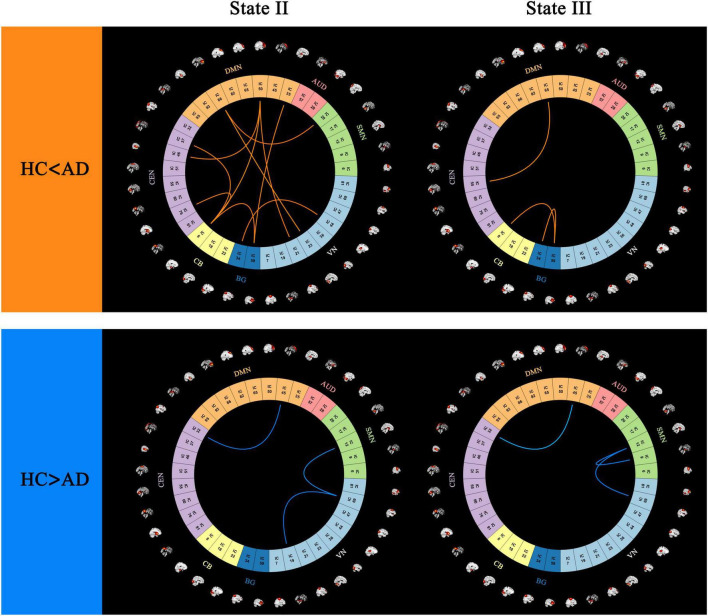
Differences in functional connectivity patterns between (AD) and (HC) groups, highlighting regions with stronger and weaker connectivity in the (AD) group (*p* < 0.05, FDR correction).

In state III, one within-network connection and two between-network connections (BG, DMN-CEN, and CB-BG) exhibited greater strength in the AD group than in the HC group. Conversely, one within-network connection and two between-network functional connections (SMN, VN-SMN, and DMN-CEN) were weaker in the AD group compared to the HC group (*p* < 0.05, FDR correction) (Figure 5).

### 3.5 Correlation between dFNC properties and clinical scores

Partial correlation analysis between temporal properties and clinical variables in patients with AD identified a significant negative correlation between the fractional windows in state III and the CAVLT-D (*r* = −0.280, *p* = 0.012) as well as the VFT (*r* = −0.248, *p* = 0.026). Additionally, the mean dwell time in state III demonstrated a negative correlation with the VFT (*r* = −0.247, *p* = 0.027). No significant correlations were observed between the temporal properties of state IV and clinical scores, nor between NPI-12 scores and any temporal properties. Detailed results are presented in Table 2.

**TABLE 2:** Significant correlations between dynamic functional connectivity temporal properties and clinical variables.

		MMSE	MoCA	NPI-12	Cognitive domains						
					CAVLT-I	CAVLT-D	CAVLT-R	VFT	DS-F	DS-B	CDT
FW in state 3	*r*	0.046	0.011	−0.202	0.173	−0.280	−0.094	−0.248	−0.043	−0.020	−0.084
	*P*	0.682	0.924	0.073	0.125	0.012*	0.405	0.026*	0.706	0.860	0.459
MDT in state 3	*r*	0.032	−0.026	−0.202	0.151	−0.133	−0.188	−0.247	−0.078	0.033	−0.055
	*P*	0.778	0.822	0.073	0.182	0.238	0.095	0.027*	0.490	0.769	0.624

A partial correlation test was also performed. Significant results are reported in bold font. *p < 0.05.

#### 3.6 Single-subject classification of individuals with AD and HC

Using a leave-one-out cross-validation approach, the linear SVM classifier achieved an accuracy of 70.37% (sensitivity = 81.54%, specificity = 53.49%, *p* = 0.005). Receiver operating characteristic (ROC) curve analysis yielded an area under the curve (AUC) of 0.68 when using the top 605 functional connections in state I to differentiate individuals with AD from HC.

**FIGURE 6:**
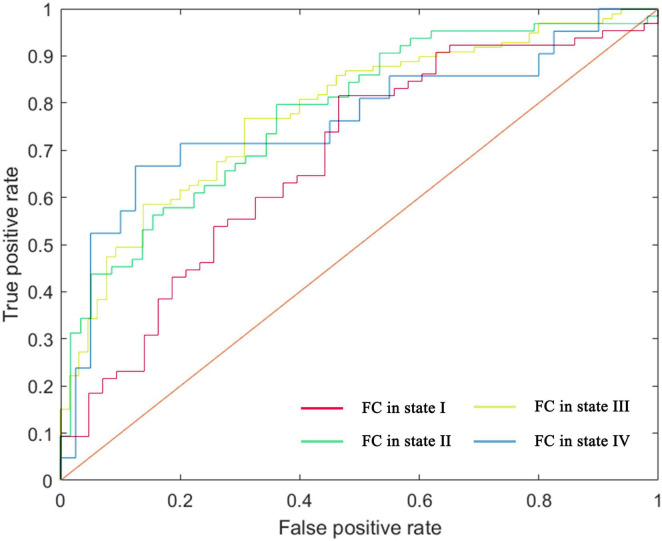
ROC curve of the classifier, depicting classification performance based on FC patterns.

For state II, classification based on the 15 highest-ranked functional connections resulted in an accuracy of 71.31% (sensitivity = 78.13%, specificity = 63.79%, *p* = 0.004), with an AUC of 0.77 on the ROC curve. In state III, the classifier achieved an accuracy of 73.17% (sensitivity = 86.87%, specificity = 52.31%, *p* = 0.001) using the 100 highest-ranked functional connections, yielding an AUC of 0.78. For state IV, classification based on the 345 highest-ranked functional connections resulted in an accuracy of 80.33% (sensitivity = 52.38%, specificity = 95.00%, *p* = 0.002), with an AUC of 0.76. Results are presented in Figure 6, Supplementary Table S4, and Supplementary Figure S13.

Performance analysis of the machine learning classifier revealed substantial differences in sensitivity and specificity among states I, III, and IV. In contrast, state II exhibited a more balanced sensitivity-specificity profile. After permutation testing, state II, which demonstrated higher AUC value, was identified as the most reliable and well-trained model. This model included 13 consensus features, primarily inter-network connections, with a few intra-network connections (Supplementary Figure S14).

## 4 Discussion

This study examined dynamic FC in individuals with AD and examined the association between dFNC properties and neuropsychological performance. The findings highlight several key observations: (1) Four distinct dFNC states were identified across all participants, with state III, characterized by sparse connectivity, occurring most frequently; (2) Temporal properties, including mean dwell time and the fractional rate of occurrence, were significantly altered in patients with AD; (3) In state III, temporal properties exhibited a significant negative correlation with cognitive performance; (4) Significant differences in FC strength between the AD and HC groups were observed in states II and III; and (5) Among all states, FC in state II demonstrated the highest classification performance for AD using the SVM classifier. These findings provide insights into the neurophysiological changes associated with AD by identifying dFNC disruptions and providing a novel perspective on its pathophysiology.

Among the four recurring dFNC states, state III was the most prevalent in the analyzed time windows, with a 10.46% higher frequency in the AD group compared to the HC group. Previous studies have indicated that this sparsely connected state represents baseline connectivity, potentially related to self-referential processing (Marusak et al., 2017). Conversely, state IV was 10.94% less frequent in the AD group than in the HC group. Unlike the sparsely connected state III, state IV exhibited strong intra-network FC within sensory networks, including the SMN and VN, alongside pronounced negative inter-network connectivity in BG-VN and BG-SMN connections.

Temporal dFNC properties differed significantly between the AD and HC groups. Individuals with AD exhibited a significantly higher proportion of fractional windows and prolonged mean dwell times in the weakly connected state III, whereas fewer fractional windows and shorter mean dwell times were observed in the highly connected state IV. These findings are consistent with previous research indicating that AD is associated with prolonged residence in weakly connected states and reduced durations in strongly connected states (Fu et al., 2019; Schumacher et al., 2019).

Recent rs-fMRI studies have linked cognitive impairment to significant changes in brain connectivity, both within localized regions and across large-scale networks. One study reported non-normal elevated FC within the CEN (Dai et al., 2019). Conversely, studies using graph theory analysis have identified both increased and decreased connectivity within networks such as the CEN and DMN, as well as disruptions or reductions in functional connections across large-scale brain networks (Zhao et al., 2019; Zhao et al., 2022).

Efficient neural communication relies on an optimal balance between local and long-distance connections. A reduction in network wiring restricts connectivity to local and long-range interactions, leading to delays in information transfer and increased metabolic energy demands. To compensate, the brain adopts a small-world topology, characterized by long-range connections that enhance network efficiency while minimizing energy expenditure (Bassett and Bullmore, 2017). In individuals with AD, resting-state networks exhibit increased vulnerability, with reduced inter-network communication and greater functional dissociation, indicating impairments in flexible brain interactions.

The relationship between dFNC temporal properties and cognitive performance was assessed, indicating a significant negative correlation between the fractional windows in state III and scores on the CAVLT-D and the VFT. A decline in CAVLT-D performance has been identified as a sensitive biomarker of memory impairment (Guo et al., 2009; Tetreault et al., 2020). Findings from previous dFNC studies in individuals with dementia and Parkinson’s disease support this association, demonstrating that a prolonged mean dwell time or an increased fractional time in sparsely connected within-network states correlates with lower cognitive scores in memory, visuospatial processing, and attention (Fiorenzato et al., 2019).

Additionally, the VFT score exhibited a negative correlation with the mean dwell time in state III. A previous study investigating AD and subcortical vascular ischemic disease reported a negative association between language function and disrupted connectivity in the parietal cortex, particularly in sparsely connected states (Fu et al., 2019). Previous research has indicated that prolonged occupancy in weakly connected states is associated with reduced cognitive reserve and decreased network efficiency (Dautricourt et al., 2022). State III was characterized by prolonged weak connectivity both within and between networks, which may contribute to slowed or uncoordinated information processing. Future studies should investigate if functional segregation, in combination with prolonged mean dwell time or increased fractional time, could serve as an early biomarker of cognitive decline in AD.

Although no significant correlation was identified between the temporal properties of state IV and cognitive function in this study, previous research on AD has indicated that strongly connected states may play a role in attentional processes, potentially facilitating information exchange between the DMN and sensory regions (Fu et al., 2019). The precuneus, a key component of the DMN, has been shown to exhibit both functional and structural impairments in patients with AD (Karas et al., 2007; Zhang et al., 2009). It is speculated that a shorter duration in this state may interfere with information exchange between the precuneus and sensory regions, potentially contributing to attentional deficits (Kim et al., 2013).

Significant differences in FC strength between states II and III were observed, involving primary sensory networks (VN, SMN, BG) and higher-order cognitive control networks (DMN, CEN, CB). These findings are consistent with previous studies indicating that resting-state FC impairments in AD predominantly affect the DMN and CEN, which are key for executive control, working memory, and attention (Brier et al., 2012; Schultz et al., 2017). Dysfunction within both primary sensory and higher-order cognitive networks is likely to contribute to cognitive decline in AD, impacting visuospatial processing, perception, and memory.

The present findings highlight disruptions in FC within and between networks across distinct states, underscoring the advantages of dFNC analysis over traditional resting-state FC approaches in capturing the temporal dynamics of connectivity (Calhoun et al., 2014). Notably, in state II, several FC differences between the AD and HC groups overlapped with consensus functional connections identified by the SVM classifier. These abnormal changes may serve as a basis for the diagnosis of AD. Furthermore, researchers often choose the unilateral or bilateral dorsolateral prefrontal cortex (DLPFC) within the CEN or the precuneus located in the DMN as therapeutic targets for transcranial magnetic stimulation (TMS) in AD patients (Saitoh et al., 2022; Cotelli et al., 2011; Koch et al., 2022). In longitudinal studies, the cross-sectional findings of this study can be used as the reference to compare dFNC changes, intra- and inter-network FC changes, and their associations with cognitive improvements after treatment.

Additionally, while a previous study using a predefined brain network template (AAL 90) reported an association between FC strength and NPI scores, no such correlation was identified in the present study (Zhao et al., 2022). This discrepancy may be attributable to differences in sample size, methodological approaches, or limitations inherent to predefined templates, which may not comprehensively capture all brain networks. The distinct connectivity patterns observed between the AD and HC groups indicate that dFNC analysis has the potential to identify functional states reflecting underlying neuropathological changes (Viviano et al., 2017).

This study has several limitations. First, the analysis did not include individuals with AD at varying disease stages, limiting the ability to examine connectivity changes associated with disease progression. Second, some participants were undergoing treatment with cholinesterase inhibitors, which may have influenced FC patterns. Third, the relatively small sample size may have reduced statistical power. Additionally, although 10 min of resting-state fMRI data is generally recommended for dFNC analysis, the present study utilized 8-min recordings. Future research with larger sample sizes, incorporating biological and genetic data, would facilitate a more comprehensive investigation of disease pathophysiology across different stages of AD.

## 5 Conclusion

This study identified abnormal dFNC patterns in individuals with AD, indicating four distinct functional connectivity states. The findings indicate that sparsely connected states are negatively correlated with cognitive function, whereas stronger connectivity states may serve as potential classifiers for distinguishing individuals with AD. These dFNC alterations provide new insights into the pathophysiology of AD and contribute to the development of improved diagnostic and prognostic approaches.

## Data Availability

The original contributions presented in this study are included in this article/Supplementary material, further inquiries can be directed to the corresponding authors.

## Footnotes

1
http://www.nitrc.org/projects/gretna

2
http://mialab.mrn.org/software/gift/
