# Correction: Altered dynamic functional network connectivity patterns in Alzheimer's disease: insights into neural dysfunction

**DOI:** 10.3389/fnagi.2025.1693649

**Published:** 2025-09-25

**Authors:** Xuerui Pang, Yi Ji, Chenyang Hu, Yulong Dai, Panpan Hu, Xingqi Wu, Kai Wang

**Affiliations:** ^1^Department of Neurology, The First Affiliated Hospital of Anhui Medical University, Hefei, China; ^2^Anhui Province Key Laboratory of Cognition and Neuropsychiatric Disorders, Hefei, China; ^3^Collaborative Innovation Center of Neuropsychiatric Disorders and Mental Health, Hefei, China; ^4^Institute of Artificial Intelligence, Hefei Comprehensive National Science Center, Hefei, China; ^5^School of Mental Health and Psychological Sciences, Anhui Medical University, Hefei, China

**Keywords:** Alzheimer's disease, dynamic functional network connectivity, functional magnetic resonance imaging, independent component analysis, network

In Table 1 of the published article, the female data of the “Gender” column in AD patients was incorrectly displayed as “28”. The corrected Table 1 appears below.

**Table 1:** Demographic and clinical data.

**Variables**	**AD patients (*n* = 100)**	**Healthy controls (*n* = 69)**	**Statistics**	***p***
**Demographic factors**				
Age (years)	65.37 ± 9.56	63.45 ± 7.33	*t* = 1.48	0.142
Gender (male/female)	48/52	28/41	*x*^2^ = 0.91	0.341
Education (years)	7.98 ± 4.78	8.42 ± 4.47	*t* = −0.60	0.550
**Psychiatric symptom**				
NPI-12	8.02 ± 11.45	1.96 ± 5.14	*t* = 4.60	0.000^**^
**General cognitive performance**				
MMSE	19.20 ± 4.97	28.50 ± 1.60	*t* = −14.99	0.000^**^
MoCA	13.32 ± 5.60	24.26 ± 3.93	*t* = −14.93	0.000^**^
**Memory performance**				
CAVLT-immediate	3.42 ± 2.04	8.49 ± 1.92	*t* = 16.01	0.000^**^
CAVLT-delay	1.33 ± 2.50	9.30 ± 2.82	*t* = 18.59	0.000^**^
CAVLT-recognition	10.70 ± 4.14	14.15 ± 1.06	*t* = −6.73	0.000^**^
**Language performance**				
VFT	9.66 ± 4.10	17.46 ± 4.03	*t* = −12.18	0.000^**^
**Attention performance**				
DS-forward	5.79 ± 1.41	6.86 ± 1.42	*t* = −4.83	0.000^**^
DS-backward	3.24 ± 1.20	4.07 ± 1.13	*t* = −4.54	0.000^**^
**Visual-spatial performance**				
CDT	1.73 ± 1.25	3.38 ± 1.00	*t* = −9.47	0.000^**^

AD, Alzheimer's disease; NPI-12, Neuropsychiatric Inventory-12; MMSE, Mini-Mental State Examination; MoCA, Montreal Cognitive Assessment; CAVLT, Chinese version of the Auditory Verbal Learning Test; VFT, Verbal Fluency Test; DS, Digit Span Test; CDT, Clock Drawing Test.

^**^p < 0.01.

The original version of this article has been updated.

