# Supplementary material for: Altered dynamic functional network connectivity patterns in Alzheimer’s disease: insights into neural dysfunction
Source: Front Aging Neurosci. 2025 Jul 23;17:1617191. doi: 10.3389/fnagi.2025.1617191 (PMC12325425; doi:10.3389/fnagi.2025.1617191)
Supplement: Supplementary file 1 [file Data_Sheet_1.docx]

***Supplementary information***

***Altered Intrinsic Brain Functional Network Dynamics in Patients with Alzheimer’s Disease***

***Supplementary methods***

**MR imaging parameters**

MRI scans were performed using a 3.0T GE scanner (GE, Healthcare, Buckinghamshire, UK). Participants were instructed to close their eyes and remain awake during the scanning procedure. Structural images were acquired using the 3D T1 BRAVO sequence with the following parameters: repetition time (TR)/echo time (TE) = 8.16/3.18 ms, flip angle = 12°, field of view = 256 mm × 256 mm, slice thickness = 1 mm, no slice gap, voxel size = 1 mm × 1 mm × 1 mm. Resting-state functional images were obtained using the Echo Planar Imaging (EPI) sequence with parameters TR/TE = 2400/30 ms, slice thickness = 3 mm, 46 continuous slices, voxel size = 3 mm × 3 mm × 3 mm, flip angle = 90°, matrix size = 64 × 64, and field of view = 192 mm × 192 mm.

**Validation analysis**

Considering the potential effect of window length on the dynamic functional network connectivity (dFNC), we performed additional validation analyses using window lengths of 15 TR, 20 TR, 25 TR, and 30 TR, respectively, with all other parameters held constant, to check the consistency and reliability of the results. During the further analysis process, if the correlation coefficient between a cluster centroid and a cluster centroid in the main analysis reaches the highest value, these two cluster centroids will be judged to be in the same state.

**Exploratory** **multivariate pattern analysis**

A concise description of machine learning classification was below: (1) At each state, a (37×36)/2 = 666 dimensional FC feature vector was generated for each subject. These intrinsic functional connections were considered as input features for classification; (2) Considering that some features are uninformative, irrelevant, or redundant for classification, reducing the number of features can not only accelerate computation but also enhance classification performance^[1,2]^. Feature selection was performed using the F-score method to improve classification performance, and this step was performed during each cross-validation^[3]^; (3) Classification was performed using a linear kernel support vector machine (SVM) classifier implemented through the LIBSVM toolbox (https://www.csie.ntu.edu.tw/~cjlin/libsvm) and the performance of the SVM classifier was evaluated using the leave-one-out cross-validation (LOOCV) strategy. The optimal hyperparameter C was selected for the linear SVM by internal five-fold cross-validation. This hyperparameter was optimised by implementing a grid search strategy (*C* = 2^-5^, 2^-4.8^, 2^-4.6^, 2^-4.4^, 2^-4.2^, … , 2^0^, 2^0.2^, 2^0.4^, 2^0.6^, 2^0.8^, … , 2^4.2^, 2^4.4^, 2^4.6^, 2^4.8^, 2^5^); (4) Features that were consistently retained across all LOOCV iterations were designated as consensus features, whereas their weights were calculated as the mean value across all validation folds. (5) Accuracy, sensitivity and specificity were calculated from the confusion matrix to quantify the performance of the SVM classifier; (6) The receiver operating characteristic (ROC) curve was constructed and the area under the curve (AUC) value was computed; (7) A non-parametric permutation test (1000 iterations) was used to assess whether the calculated classification accuracy was statistically significant. The result was considered significant if the accuracy of all the permutations exceeded the non-permutation value in less than 5% of cases (p < 0.05).

***Supplementary results***

Additional validation analysis with a window size of 15 TR presented that the main results remain unchanged. Specifically, four dynamic FC states were also identified under this window size across all subjects (Figure S9). State II under 15 TR window size and State I under 22 TR window size (*r* = 0.9993), State III under 15 TR window size and State II under 22 TR window size (*r* = 0.9981), State I under 15 TR window size and State III under 22 TR window size (*r* = 0.9993), and State IV under 15 TR window size and State IV under 22 TR window size (*r* = 0.9996) showed similar characterization of dynamic FC states (Table S2). We discovered that the reproducibility of the main results was achievable. This was because the significant findings on the temporal properties of the main analysis persisted in the validation analysis.

Additional validation analysis with a window size of 20 TR presented that the main results remain unchanged. Specifically, four dynamic FC states were also identified under this window size across all subjects (Figure S10). State IV under 20 TR window size and State I under 22 TR window size (*r* = 0.9999), State II under 20 TR window size and State II under 22 TR window size (*r* = 0.9998), State IV under 20 TR window size and State III under 22 TR window size (*r* = 0.9999), and State I under 20 TR window size and State IV under 22 TR window size (*r* = 1.0000) showed similar characterization of dynamic FC states (Table S2). We discovered that the reproducibility of the main results was achievable. This was because the significant findings on the temporal properties of the main analysis persisted in the validation analysis.

Additional validation analysis with a window size of 25 TR presented that the main results remain unchanged. Specifically, four dynamic FC states were also identified under this window size across all subjects (Figure S11). State IV under 25 TR window size and State I under 22 TR window size (*r* = 0.9345), State III under 25 TR window size and State II under 22 TR window size (*r* = 0.9462), State I under 25 TR window size and State III under 22 TR window size (*r* = 0.9928), and State II under 25 TR window size and State IV under 22 TR window size (*r* = 0.9523) showed similar characterization of dynamic FC states (Table S3). We discovered that the reproducibility of the main results was achievable. This was because the significant findings on the temporal properties of the main analysis persisted in the validation analysis.

Additional validation analysis with a window size of 30 TR presented that the main results remain unchanged. Specifically, four dynamic FC states were also identified under this window size across all subjects (Figure S12). State II under 30 TR window size and State I under 22 TR window size (*r* = 0.9223), State IV under 30 TR window size and State II under 22 TR window size (*r* = 0.9404), State III under 30 TR window size and State III under 22 TR window size (*r* = 0.9901), and State I under 30 TR window size and State IV under 22 TR window size (*r* = 0.9470) showed similar characterization of dynamic FC states (Table S3). We discovered that the reproducibility of the main results was achievable. This was because the significant findings on the temporal properties of the main analysis persisted in the validation analysis.

**Table S1.** Peak activation information of 37 independent components

| **Intrinsic connectivity network** | **The number of IC** | **MNI peak coordinate** | | | **Dynamic Range** | **PowerLF/**  **PowerH** |
| --- | --- | --- | --- | --- | --- | --- |
|  |  | *X* | *Y* | *Z* |  |  |
| **Visual network (VN)**  **Sensorimotor network (SMN)**  **Auditory network (AUD)**  **Default mode network (DMN)**  **Cognitive executive network (CEN)**  **Cerebellar network (CB)**  **Basal ganglia network (BG)** | IC 07  IC 19  IC 21  IC 23  IC 36  IC 44  IC 47  IC 69  IC 81  IC 06  IC 09  IC 12  IC 17  IC 20  IC 28  IC 57  IC 35  IC 45  IC 50  IC 65  IC 82  IC 86  IC 93  IC 95  IC 32  IC 37  IC 46  IC 51  IC 55  IC 60  IC 76  IC 91  IC 08  IC 18  IC 22  IC 34  IC 59 | 29.5  2.5  9.5  -21.5  51.5  -5.5  24.5  -5.5  -11.5  57.5  38.5  -35.5  26.5  0.5  60.5  -59.5  2.5  -39.5  -41.5  0.5  0.5  59.5  42.5  -0.5  41.5  0.5  30.5  -51.5  45.5  32.5  50.5  -50.5  -32.5  -2.5  -6.5  0.5  23.5 | -96.5  -96.5  -98.5  -81.5  -71.5  -75.5  -77.5  -86.5  -53.5  -5.5  -21.5  -23.5  -53.5  -38.5  2.5  -29.5  -74.5  -60.5  -72.5  47.5  -62.5  -47.5  -69.5  57.5  -57.5  24.5  62.5  15.5  42.5  50.5  15.5  20.5  -83.5  -66.5  -54.5  -17.5  9.5 | -2.5  14.5  -0.5  -15.5  3.5  9.5  -14.5  39.5  -0.5  24.5  68.5  69.5  71.5  72.5  -3.5  15.5  51.5  57.5  41.5  50.5  35.5  36.5  45.5  8.5  59.5  62.5  3.5  30.5  9.5  35.5  39.5  -5.5  -23.5  -21.5  -41.5  3.5  -0.5 | 0.050  0.044  0.049  0.051  0.051  0.044  0.046  0.051  0.039  0.045  0.051  0.050  0.045  0.048  0.049  0.039  0.050  0.045  0.046  0.045  0.049  0.046  0.045  0.033  0.044  0.036  0.043  0.040  0.034  0.045  0.044  0.029  0.052  0.034  0.032  0.036  0.034 | 4.024  2.637  3.508  3.980  3.860  2.679  2.710  4.031  1.860  2.862  4.073  3.781  2.738  3.359  2.700  1.349  3.780  2.455  2.720  2.463  3.277  2.517  2.804  1.128  2.540  1.556  2.113  1.772  1.267  2.110  2.319  0.998  3.391  1.092  0.845  1.273  1.230   \|  \| \| --- \| |

***Note:*** the coordinates are peak voxel coordinates of the one-sample t-test results for each independent component spatial maps of all subjects. IC, independent component; MNI, Montreal Neurological Institute; *I*_q_, quality index; *Power*_LF_/*Power*_HF_, low frequency (LF) to high frequency (HF) power ratio.

| **Table S2.** Correlation coefficients between pairs of cluster centroids under different window sizes | | | | | | | | | | |
| --- | --- | --- | --- | --- | --- | --- | --- | --- | --- | --- |
| **Window size=22TR** |  | **Window size=15TR** | | | |  | **Window size=20TR** | | | |
|  |  | **State I** | **State II** | **State III** | **State IV** |  | **State I** | **State II** | **State III** | **State IV** |
| **S**tate I |  | 0.7438 | 0.9993* | 0.5630 | 0.6993 |  | 0.7042 | 0.5786 | 0.9999 | 0.9999* |
| State II |  | 0.8123 | 0.5653 | 0.9981* | 0.6416 |  | 0.6520 | 0.9998* | 0.5773 | 0.8136 |
| State III |  | 0.9993* | 0.7292 | 0.7997 | 0.5840 |  | 0.5945 | 0.8090 | 0.7393 | 0.9999* |
| State IV |  | 0.6098 | 0.7015 | 0.6346 | 0.9996* |  | 1.0000* | 0.6511 | 0.7054 | 0.6024 |

* Indicates the highest similarity of state centroids under each pair of window sizes.

| **Table S3.** Correlation coefficients between pairs of cluster centroids under different window sizes | | | | | | | | | | |
| --- | --- | --- | --- | --- | --- | --- | --- | --- | --- | --- |
| **Window size=22TR** |  | **Window size=25TR** | | | |  | **Window size=30TR** | | | |
|  |  | **State I** | **State II** | **State III** | **State IV** |  | **State I** | **State II** | **State III** | **State IV** |
| State I |  | 0.8123 | 0.5689 | 0.6454 | 0.9345* |  | 0.5673 | 0.9223* | 0.8229 | 0.6575 |
| State II |  | 0.7888 | 0.7537 | 0.9462* | 0.5769 |  | 0.7630 | 0.5851 | 0.7819 | 0.9404* |
| State III |  | 0.9928* | 0.5946 | 0.8631 | 0.6666 |  | 0.6015 | 0.6642 | 0.9901* | 0.8727 |
| State IV |  | 0.6242 | 0.9523* | 0.5124 | 0.8895 |  | 0.9470* | 0.9052 | 0.6276 | 0.5124 |

* Indicates the highest similarity of state centroids under each pair of window sizes.

**Table S4.** Classification performance of the linear support vector machine classifier

|  | **Optimal feature size** | **Accuracy (%)** | **Sensitivity (%)** | **Specificity(%)** | **AUC** | ***p* value** |
| --- | --- | --- | --- | --- | --- | --- |
| FC in State I | 605 | 70.37 | 81.54 | 53.49 | 0.68 | 0.005 |
| FC in State II | 15 | 71.31 | 78.13 | 63.79 | 0.77 | 0.004 |
| FC in State III | 100 | 73.17 | 86.87 | 52.31 | 0.78 | 0.001 |
| FC in State IV | 345 | 80.33 | 52.38 | 95.00 | 0.76 | 0.002 |

FC, functional connectivity; AUC, area under the curve

**
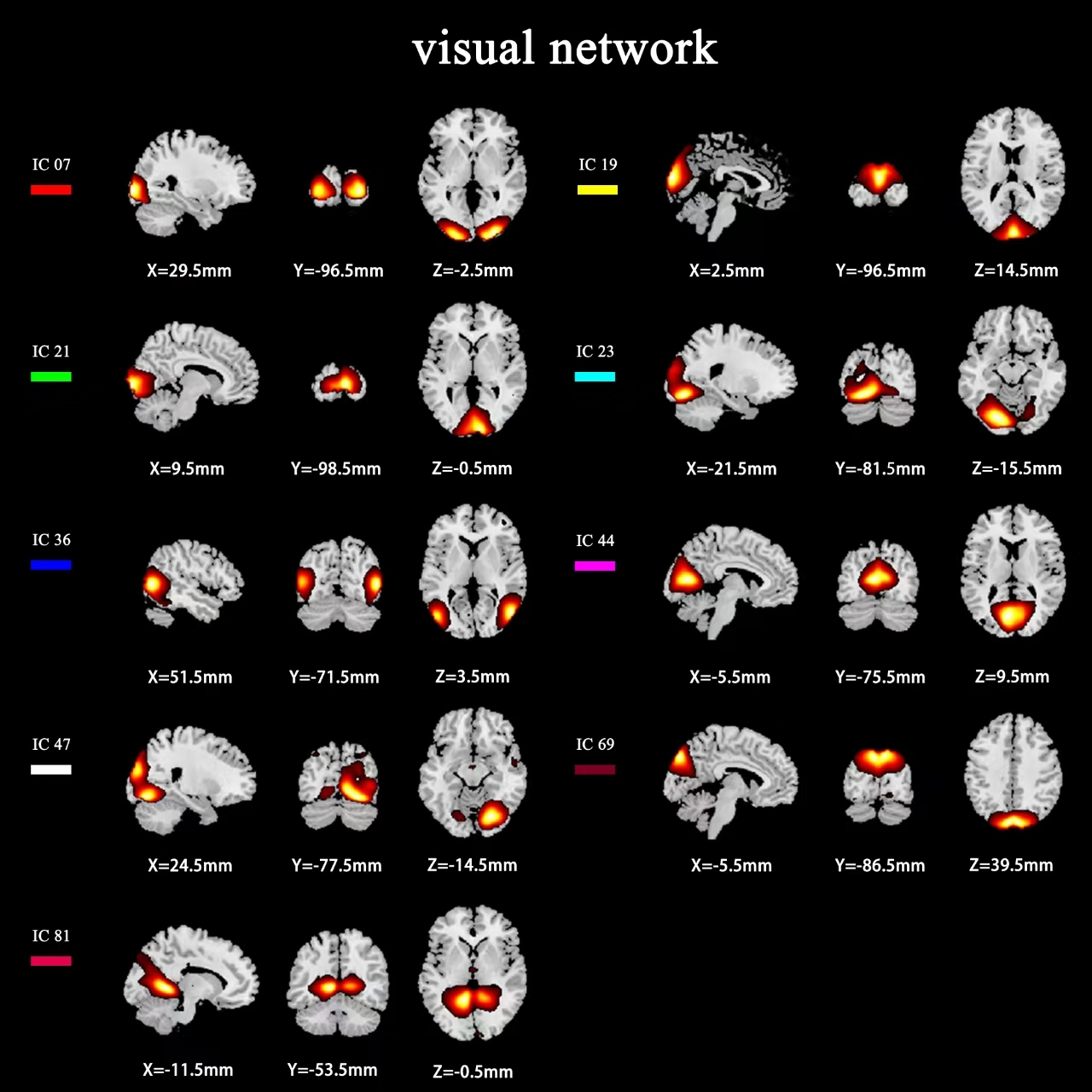
**

**Figure S1.** Spatial maps of intrinsic connectivity networks in visual network. The colored legend next to each IC matches the overlay color of the spatial map in Figure 1. The sagittal, coronal, and axial slices are presented when the t-statistic reaches its maximum value for the clusters that exhibit the highest level of activation.

**
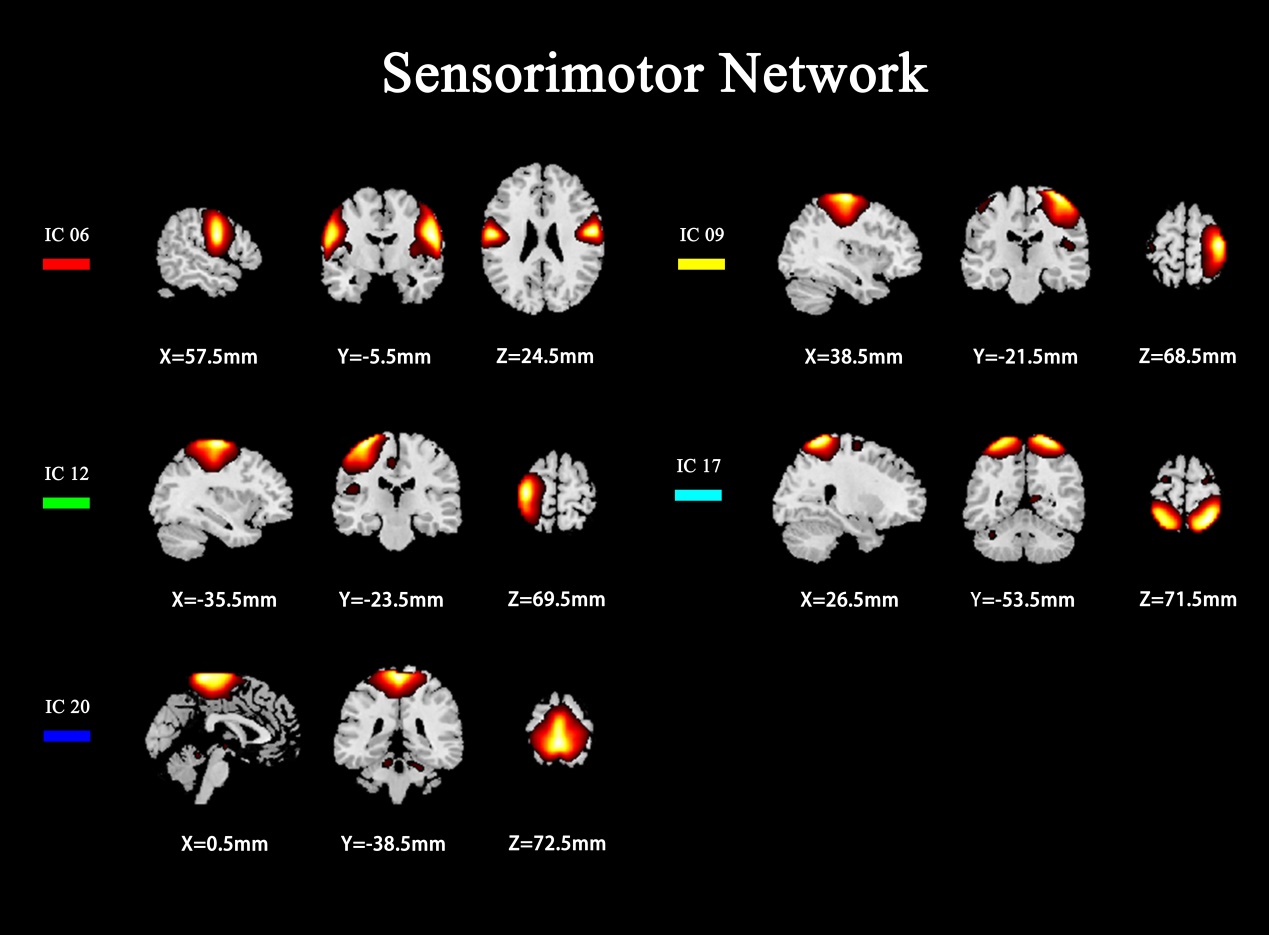
Figure S2.** Spatial maps of intrinsic connectivity networks in sensorimotor network. The colored legend next to each IC matches the overlay color of the spatial map in Figure 1. The sagittal, coronal, and axial slices are presented when the t-statistic reaches its maximum value for the clusters that exhibit the highest level of activation.

**
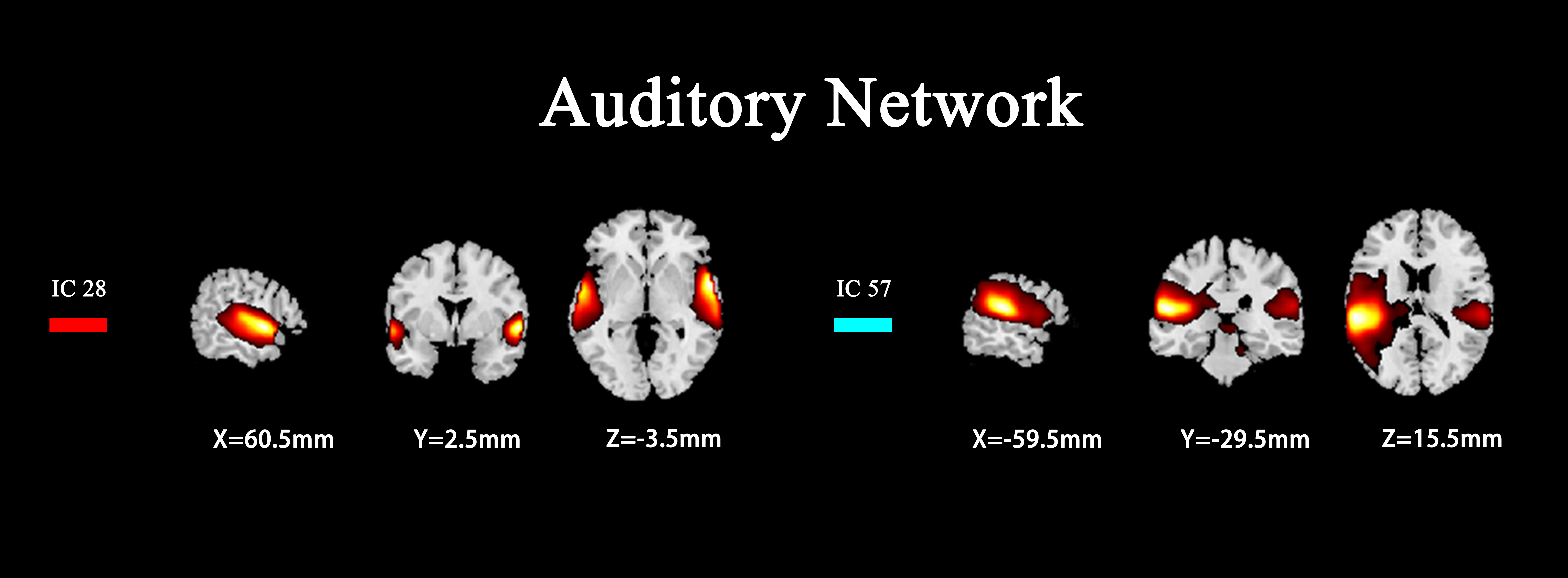
Figure S3.** Spatial maps of intrinsic connectivity networks in auditory network. The colored legend next to each IC matches the overlay color of the spatial map in Figure 1. The sagittal, coronal, and axial slices are presented when the t-statistic reaches its maximum value for the clusters that exhibit the highest level of activation.

**
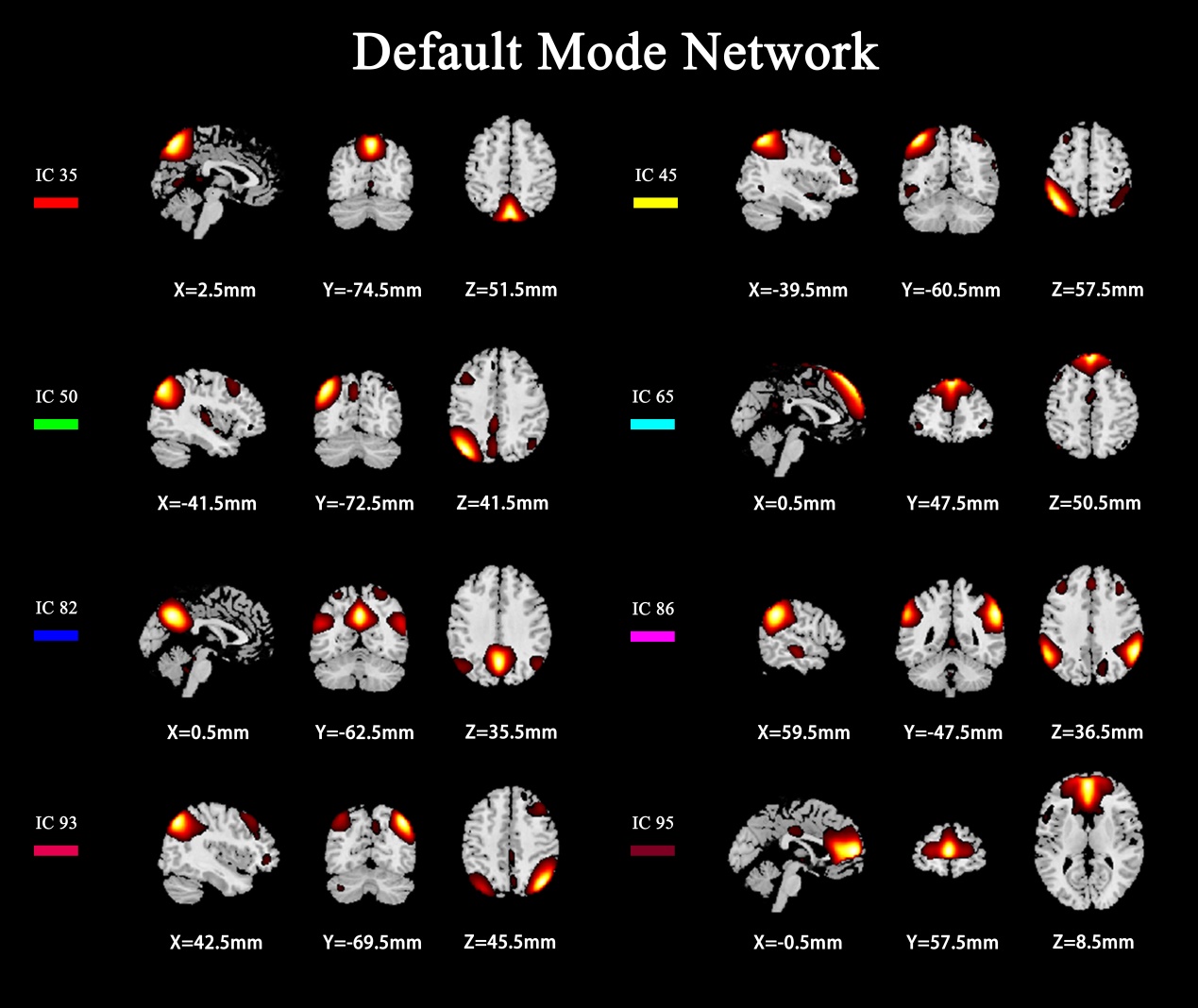
Figure S4.** Spatial maps of intrinsic connectivity networks in default mode network network. The colored legend next to each IC matches the overlay color of the spatial map in Figure 1. The sagittal, coronal, and axial slices are presented when the t-statistic reaches its maximum value for the clusters that exhibit the highest level of activation.

**
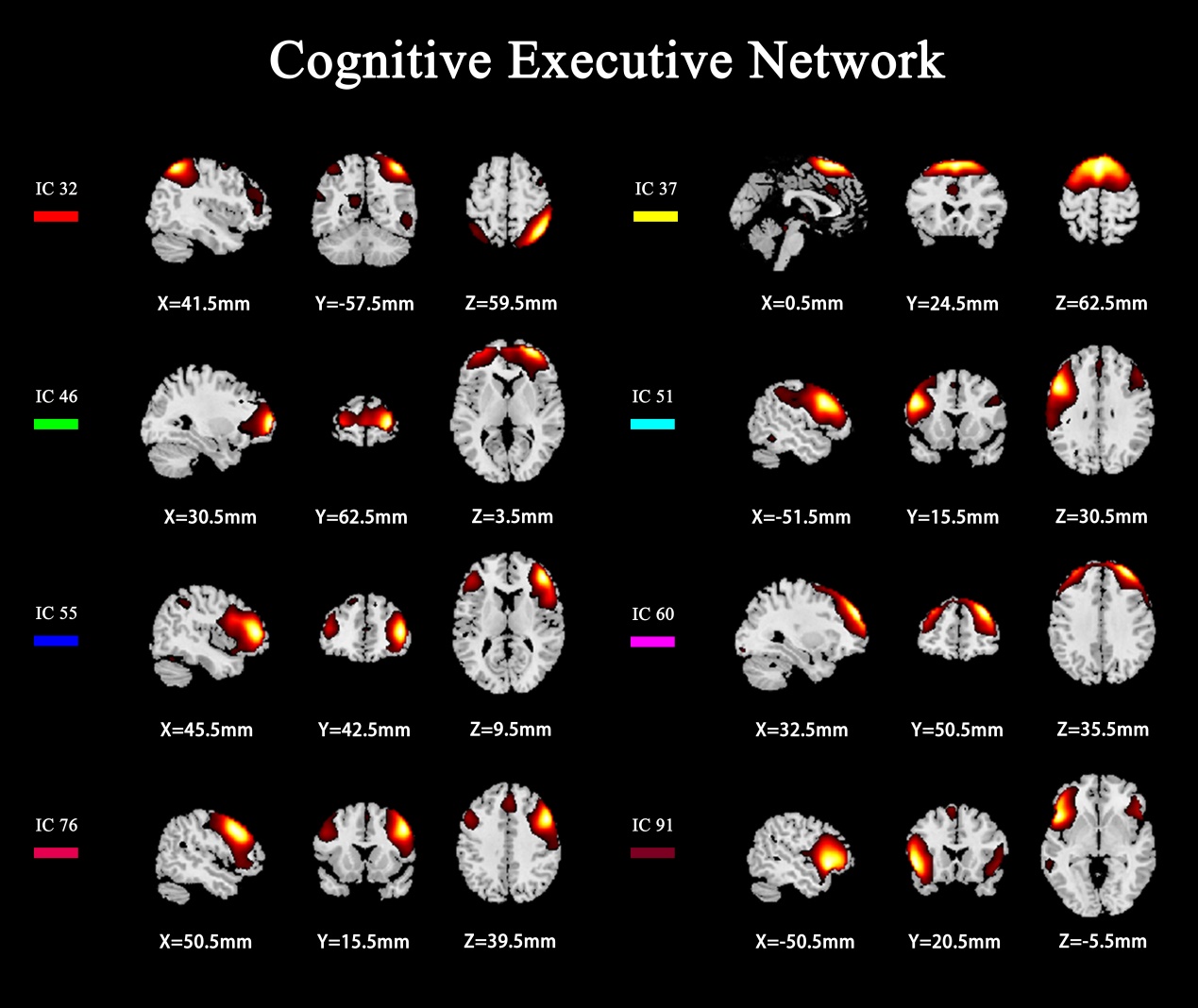
Figure S5.** Spatial maps of intrinsic connectivity networks in cognitive executive network. The colored legend next to each IC matches the overlay color of the spatial map in Figure 1. The sagittal, coronal, and axial slices are presented when the t-statistic reaches its maximum value for the clusters that exhibit the highest level of activation.

**
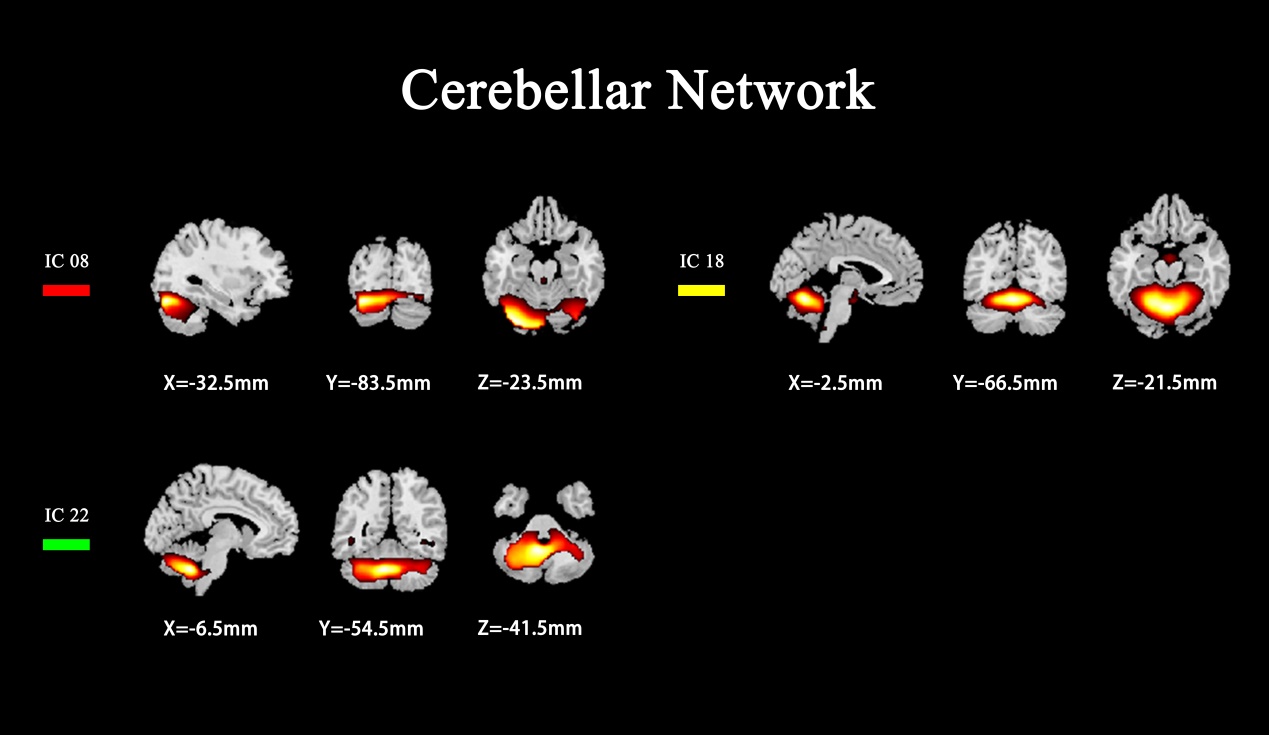
Figure S6.** Spatial maps of intrinsic connectivity networks in cerebellar network. The colored legend next to each IC matches the overlay color of the spatial map in Figure 1. The sagittal, coronal, and axial slices are presented when the t-statistic reaches its maximum value for the clusters that exhibit the highest level of activation.

**
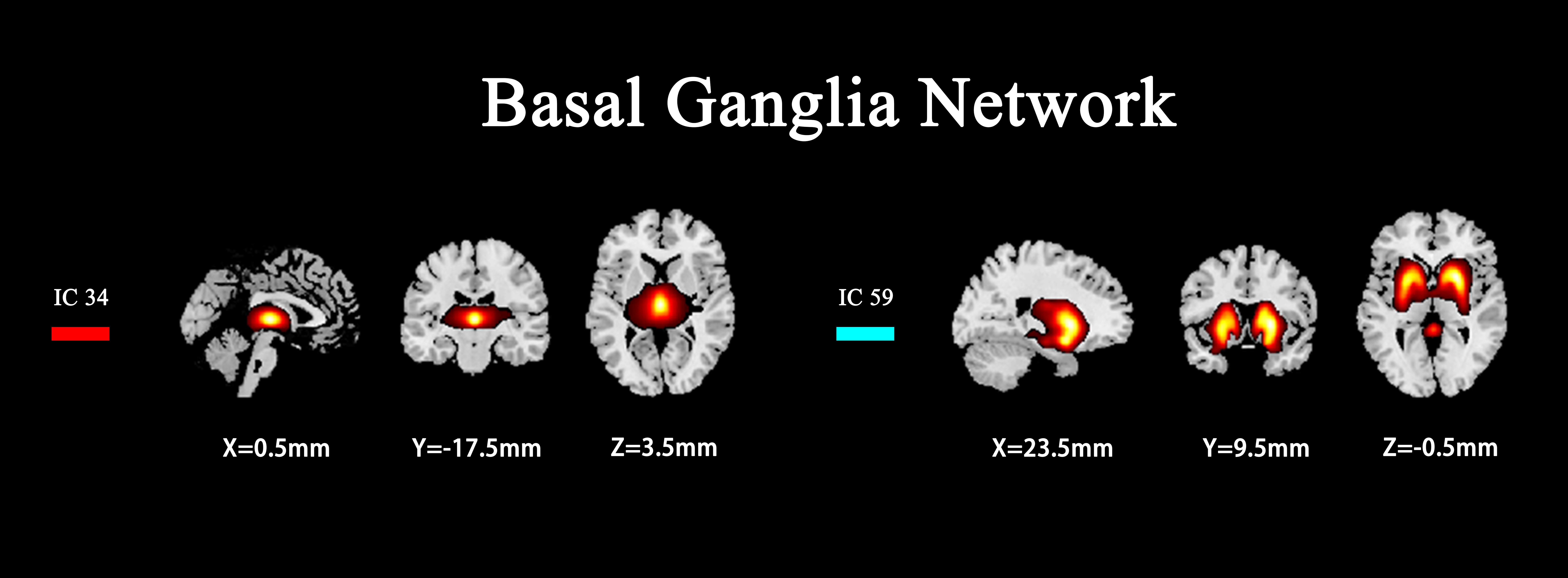
**

**Figure S7.** Spatial maps of intrinsic connectivity networks in basal ganglia network. The colored legend next to each IC matches the overlay color of the spatial map in Figure 1. The sagittal, coronal, and axial slices are presented when the t-statistic reaches its maximum value for the clusters that exhibit the highest level of activation.

**
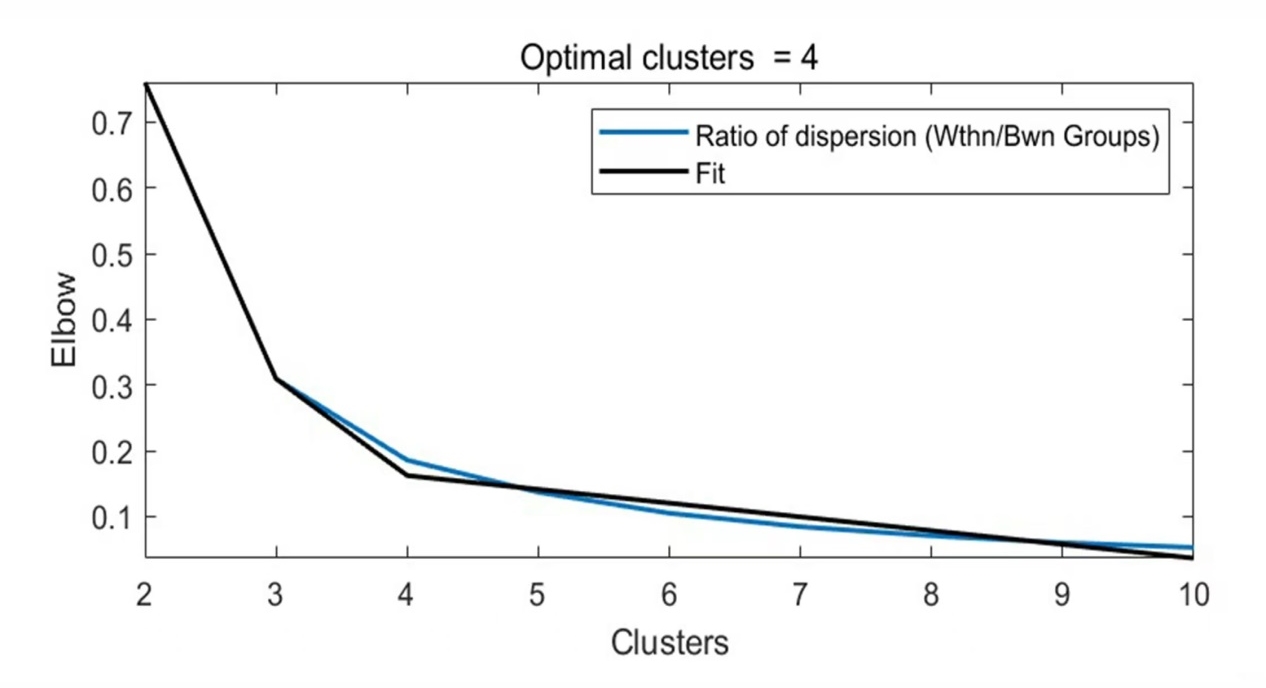
Figure S8.** K‐means clustering analysis assessing the reoccurring dynamic functional connectivity patterns. The optimal cluster number was four, which was determined using the elbow criterion.


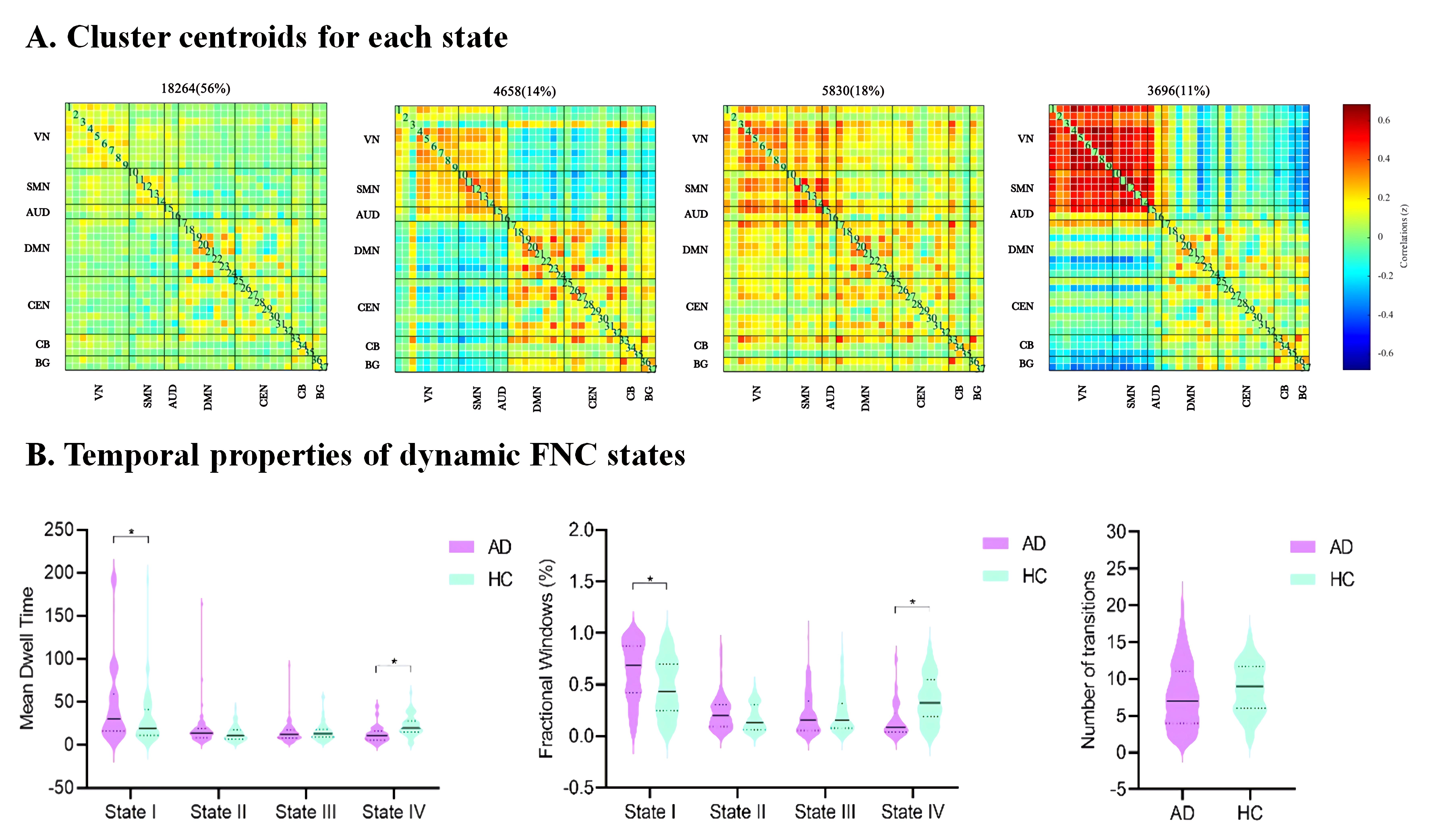


**Figure S9.** Validation analysis results under the window size of 15TR. A. Cluster centroids for each state for all participants. B. Group comparison of temporal properties of dynamic functional connectivity states. The Fractional windows, Mean dwell time and number of transitions are shown using violin plots for the AD group (purple) and HC group (blue). Horizontal lines indicate group median (solid lines) and upper and lower quartiles (dashed lines). Asterisk represents p<0.05.


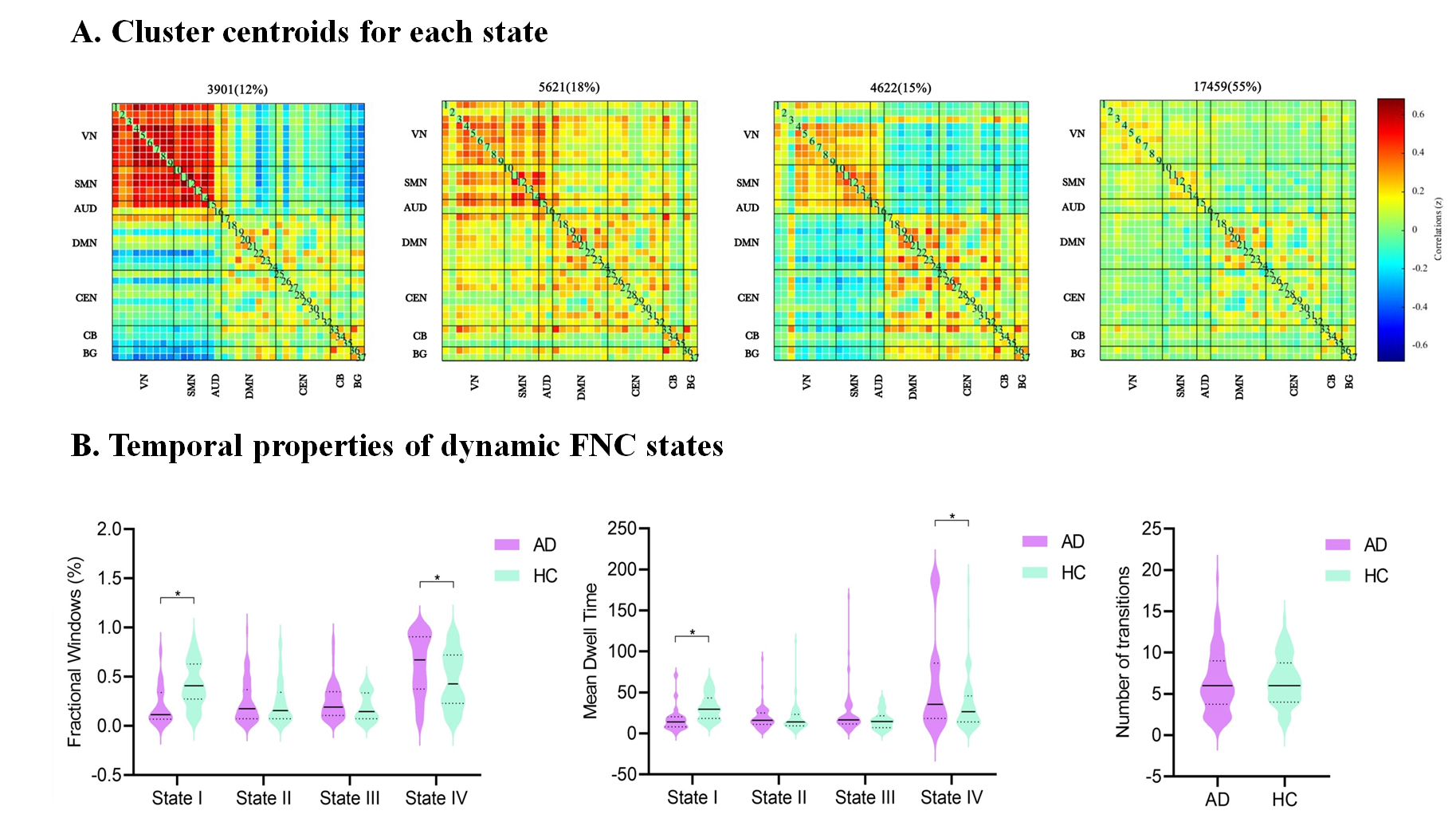


**Figure S10.** Validation analysis results under the window size of 20TR. A. Cluster centroids for each state for all participants. B. Group comparison of temporal properties of dynamic functional connectivity states. The Fractional windows, Mean dwell time and number of transitions are shown using violin plots for the AD group (purple) and HC group (blue). Horizontal lines indicate group median (solid lines) and upper and lower quartiles (dashed lines). Asterisk represents p<0.05.


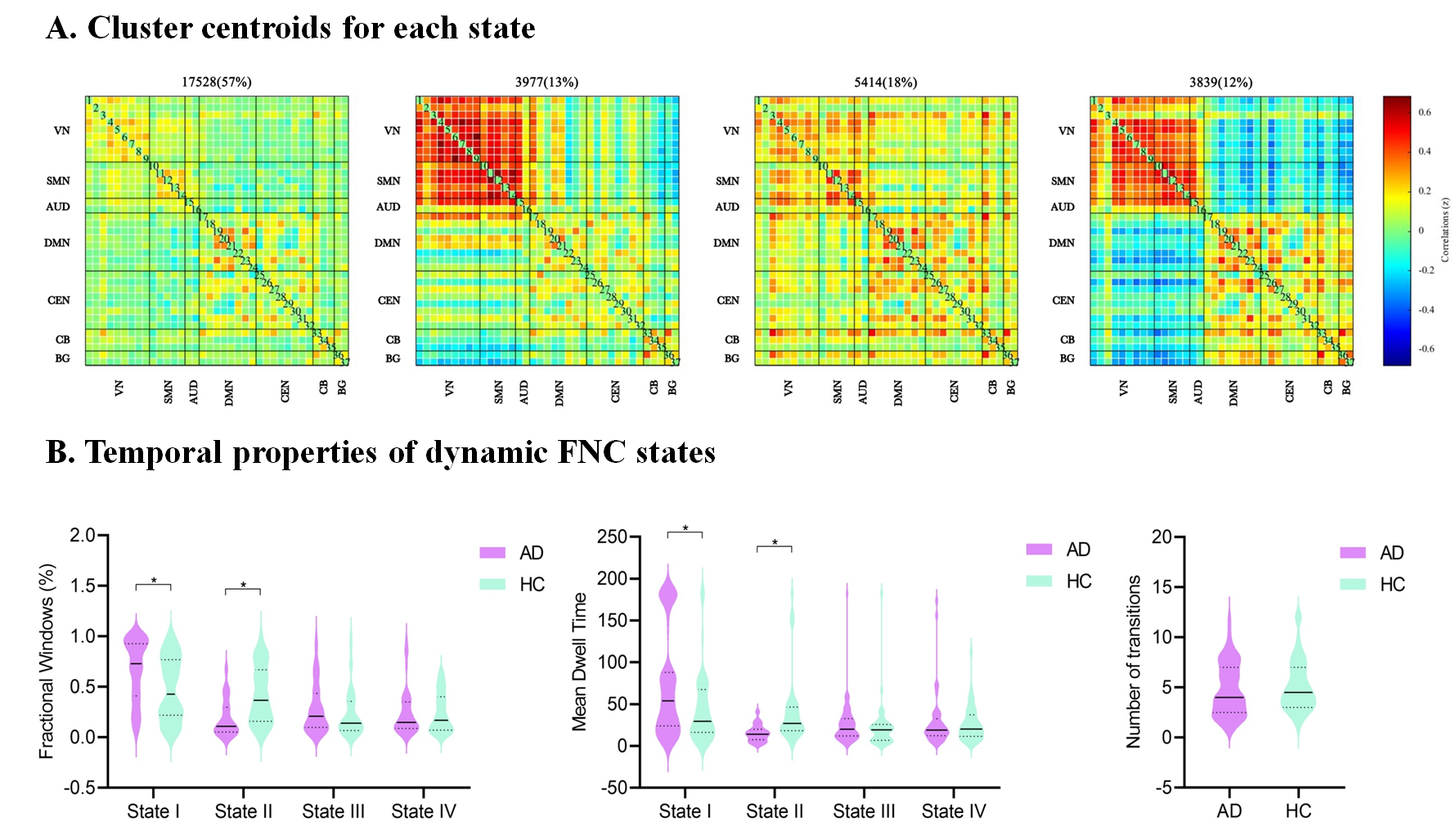


**Figure S11.** Validation analysis results under the window size of 25TR. A. Cluster centroids for each state for all participants. B. Group comparison of temporal properties of dynamic functional connectivity states. The Fractional windows, Mean dwell time and number of transitions are shown using violin plots for the AD group (purple) and HC group (blue). Horizontal lines indicate group median (solid lines) and upper and lower quartiles (dashed lines). Asterisk represents p<0.05.


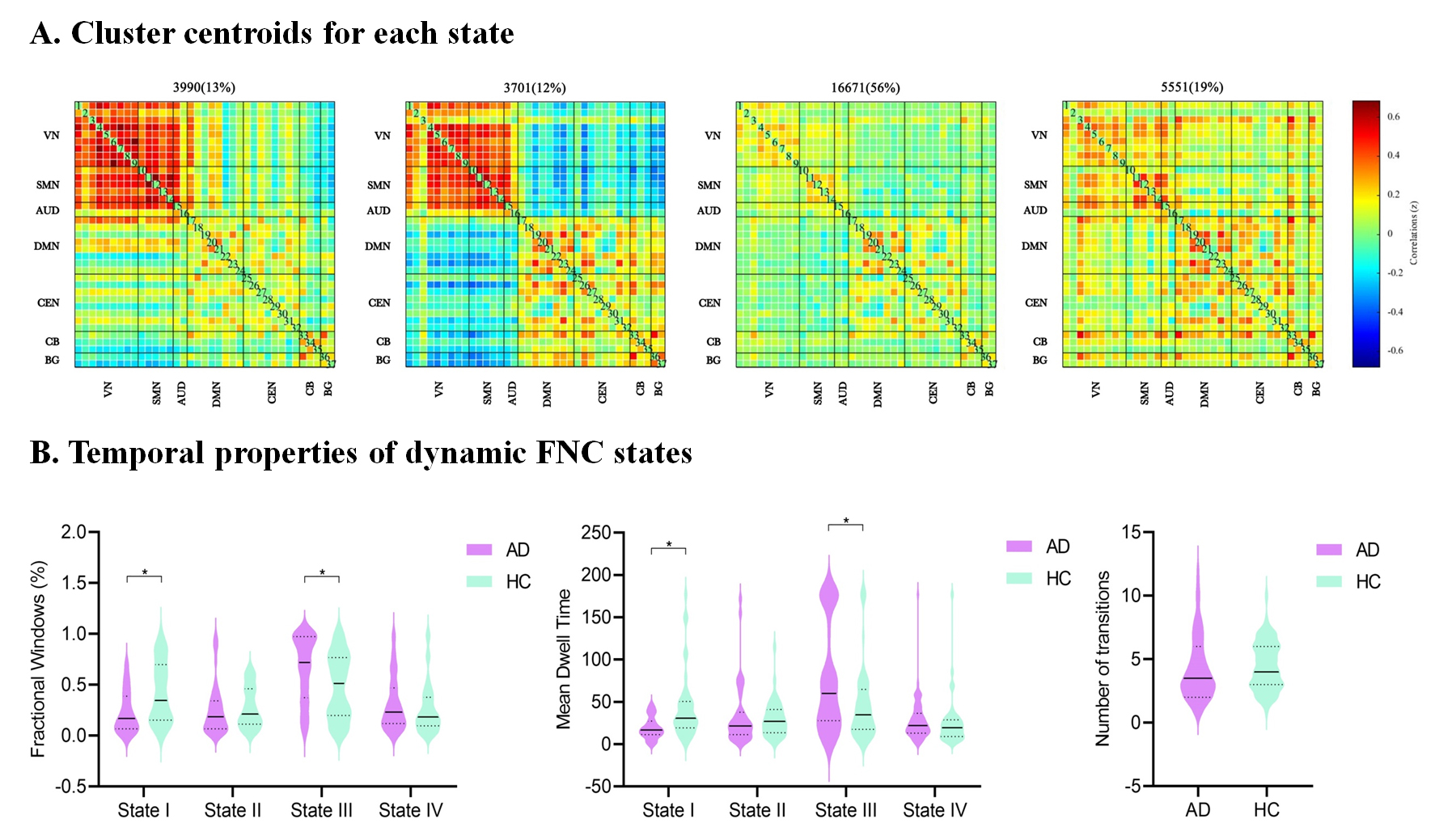


**Figure S12.** Validation analysis results under the window size of 30TR. A. Cluster centroids for each state for all participants. B. Group comparison of temporal properties of dynamic functional connectivity states. The Fractional windows, Mean dwell time and number of transitions are shown using violin plots for the AD group (purple) and HC group (blue). Horizontal lines indicate group median (solid lines) and upper and lower quartiles (dashed lines). Asterisk represents p<0.05.

**
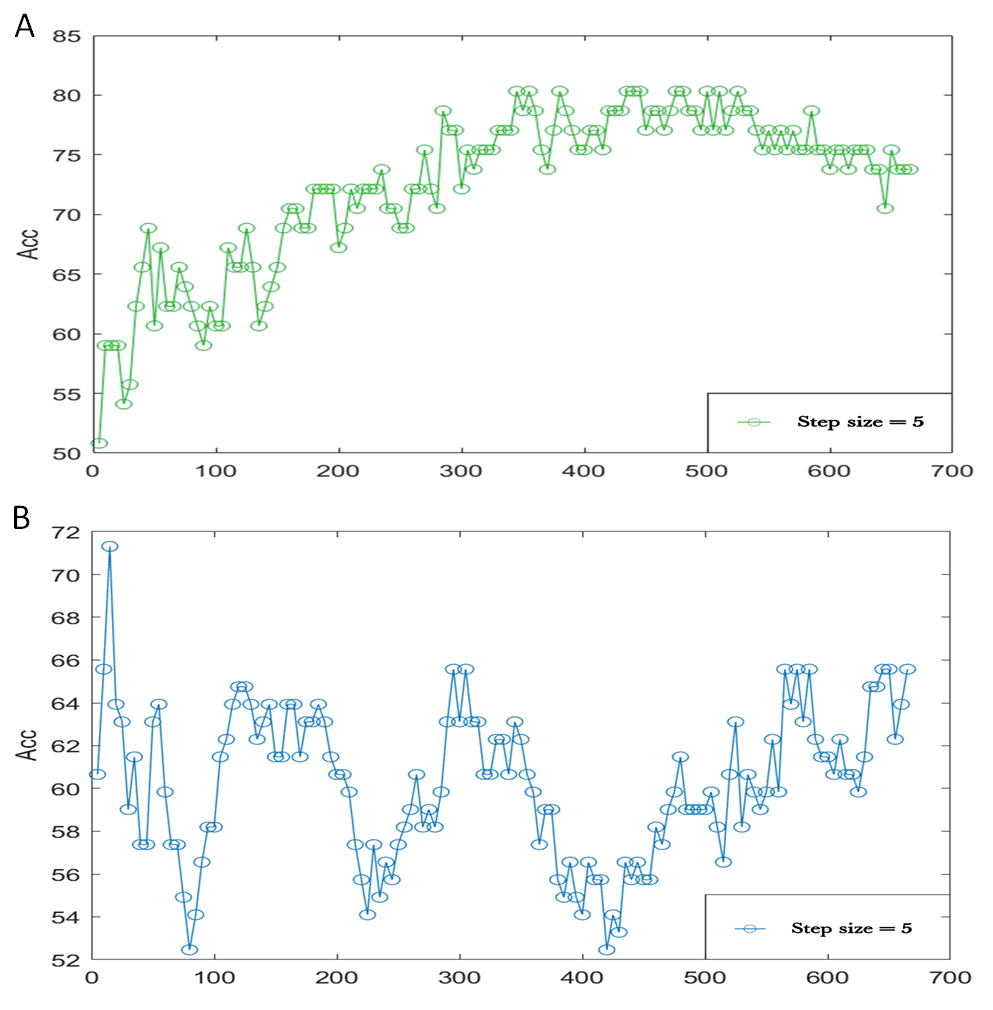
**

**
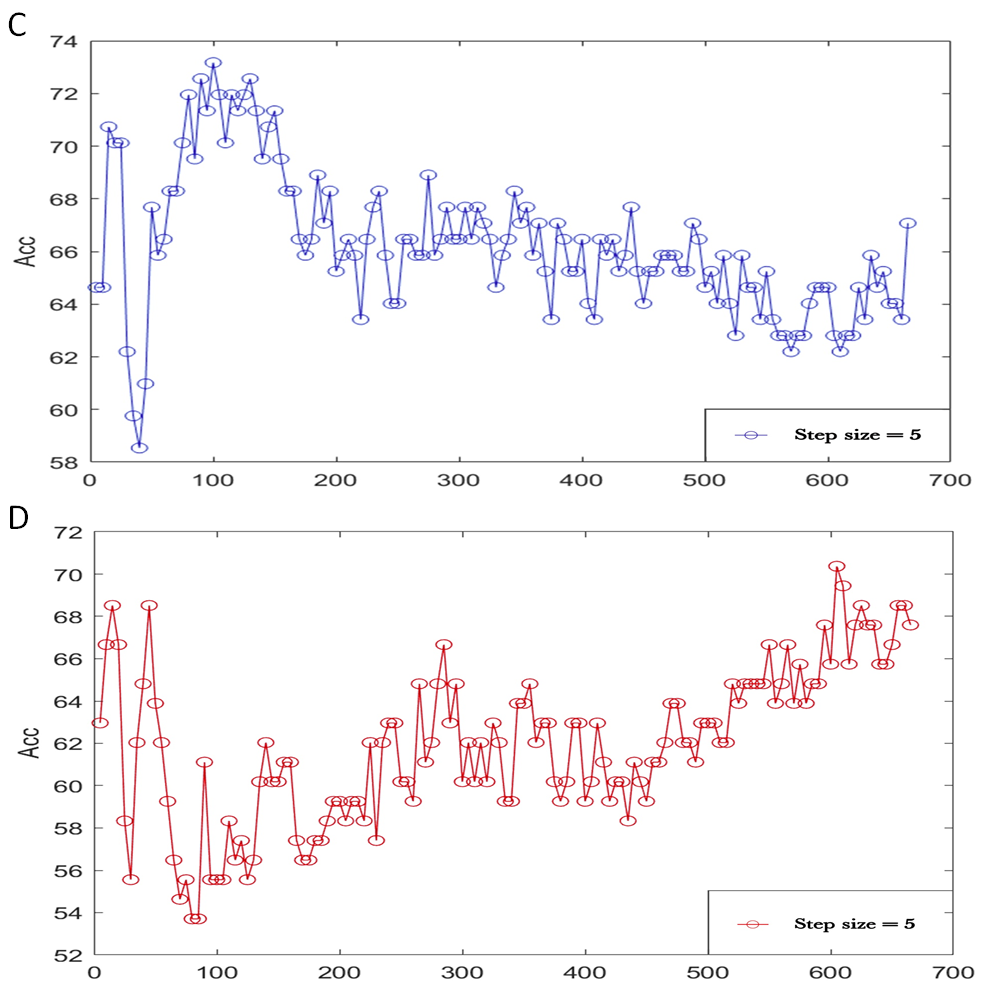
**

**Figure S13.** Classification accuracy under different number of functional connections retained in state I A, state II B, state III C and state IV D. The connections were ranked according to F scores in descending order. ACC：accuracy

**
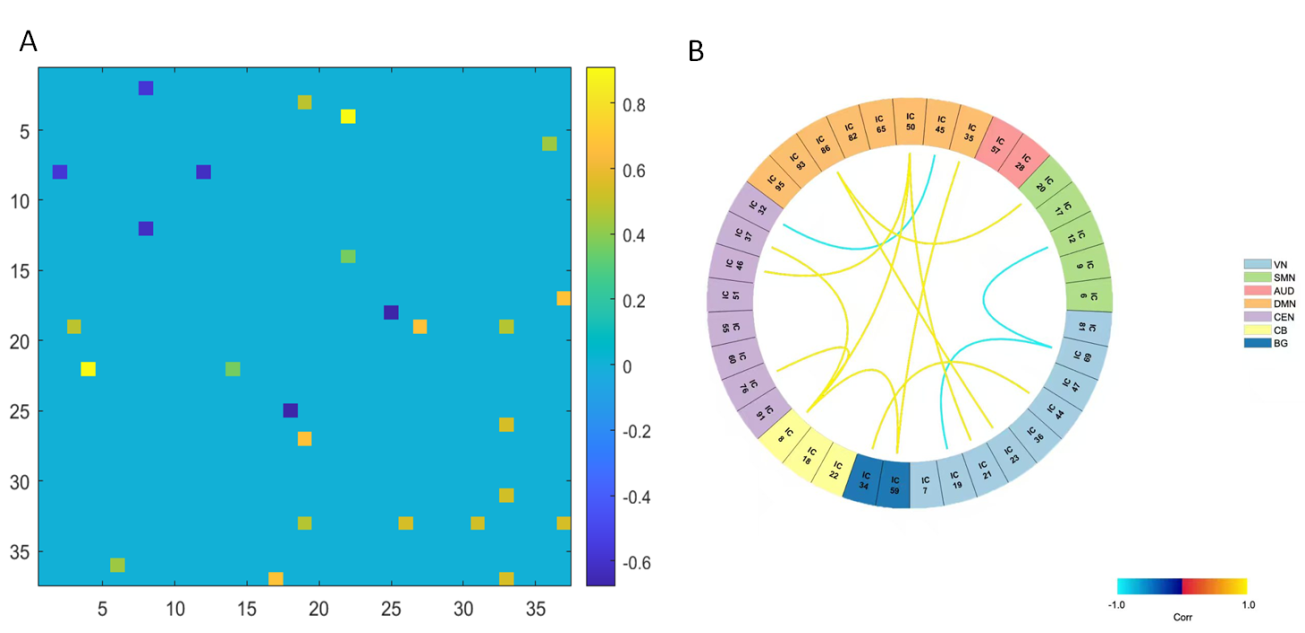
**

**Figure S14.** Consensus functional connections in State II. A. Matrix shows feature weights of the consensus functional connections. B. The connectogram of group differences in FC is visualized using a circular graph. Each square color represents one of the seven networks. Yellow lines represent positive weights and blue lines represent negative weights for classification. VN, visual network; SMN, sensorimotor network; AUD, auditory network; DMN, default mode network; CEN, cognitive executive network; CB, cerebellar network; BG, basal ganglia network

**References**

[1] Dosenbach NU, Nardos B, Cohen AL, Fair DA, Power JD, Church JA, Nelson SM, Wig GS, Vogel AC, Lessov-Schlaggar CN, Barnes KA, Dubis JW, Feczko E, Coalson RS, Pruett JR Jr, Barch DM, Petersen SE, Schlaggar BL. Prediction of individual brain maturity using fMRI. Science. 2010 Sep 10;329(5997):1358-61. doi: 10.1126/science.1194144. Erratum in: Science. 2010 Nov 5;330(6005):756. PMID: 20829489; PMCID: PMC3135376.

[2] Pereira F, Mitchell T, Botvinick M. Machine learning classifiers and fMRI: a tutorial overview. Neuroimage. 2009 Mar;45(1 Suppl):S199-209. doi: 10.1016/j.neuroimage.2008.11.007. Epub 2008 Nov 21. PMID: 19070668; PMCID: PMC2892746.

[3] Chen YW, Lin CJ. Combining SVMs with Various Feature Selection Strategies. In: Guyon, I., Nikravesh, M., Gunn, S., Zadeh, L.A. (eds) Feature Extraction: Foundations and Applications. Berlin, Heidelberg: Springer Berlin Heidelberg; 2006. p. 315-24.
